# Activation and identification of five clusters for secondary metabolites in *Streptomyces albus* J1074

**DOI:** 10.1111/1751-7915.12116

**Published:** 2014-03-04

**Authors:** Carlos Olano, Ignacio García, Aranzazu González, Miriam Rodriguez, Daniel Rozas, Julio Rubio, Marina Sánchez-Hidalgo, Alfredo F Braña, Carmen Méndez, José A Salas

**Affiliations:** Departamento de Biología Funcional e Instituto Universitario de Oncología del Principado de Asturias (I.U.O.P.A), Universidad de OviedoOviedo, Spain

## Abstract

*Streptomyces albus* J1074 is a streptomycete strain widely used as a host for expression of secondary metabolite gene clusters. Bioinformatic analysis of the genome of this organism predicts the presence of 27 gene clusters for secondary metabolites. We have used three different strategies for the activation of some of these silent/cryptic gene clusters in *S. albus* J1074: two hybrid polyketide-non-ribosomal peptides (PK-NRP) (antimycin and 6-*epi*-alteramides), a type I PK (candicidin), a non-ribosomal peptides (NRP) (indigoidine) and glycosylated compounds (paulomycins). By insertion of a strong and constitutive promoter in front of selected genes of two clusters, production of the blue pigment indigoidine and of two novel members of the polycyclic tetramate macrolactam family (6-*epi*-alteramides A and B) was activated. Overexpression of positive regulatory genes from the same organism also activated the biosynthesis of 6-*epi*-alteramides and heterologous expression of the regulatory gene *pimM* of the pimaricin cluster activated the simultaneous production of candicidins and antimycins, suggesting some kind of cross-regulation between both clusters. A cluster for glycosylated compounds (paulomycins) was also identified by comparison of the high-performance liquid chromatography profiles of the wild-type strain with that of a mutant in which two key enzymes of the cluster were simultaneously deleted.

## Introduction

Actinomycetes are Gram-positive bacteria belonging to the phylum *Actinobacteria*, one of the largest taxonomic groups within the domain Bacteria (Gao and Gupta, 2012). Filamentous actinomycetes are characterized by a complex life cycle of morphological differentiation that starts with the germination of a spore to give a substrate mycelium and later an aerial mycelium, followed then by a sporulation stage (Chater, 1998). *Actinobacteria* are widely distributed in terrestrial, especially soil, and aquatic ecosystems (McCarthy and Williams, 1992; Stach and Bull, 2005). They are important for soil formation by decomposing and recycling complex mixtures of polymers in dead plant, animal and fungal materials using extracellular enzymes (McCarthy and Williams, 1992). They also exhibit diverse physiological and metabolic properties, such as the production of volatile substances (Gust *et al*., 2003) and a wide variety of secondary metabolites, many of which are potent antibiotic, antifungal, antitumor, immunosuppressant, antiviral or antiparasitic agents. Among these compounds, some structural types such as polyene macrolides, large-membered macrolides, anthracyclines, polyethers, cyclopolylactones, aminoglycosides, streptothricins, actinomycins and quinoxaline peptides are produced almost exclusively by actinomycetes. In addition, glycopeptides and orthosomycins are mainly produced by non-*Streptomyces* species (Bérdy, 2012). This has turned actinomycetes into the primary bioactive metabolite-producing organisms exploited by the pharmaceutical industry and has prompted the study of these microorganisms at all levels: taxonomy, genetics and physiology (Hopwood, 1999).

Since the development of recombinant DNA technology, an increasing number of biosynthesis gene clusters for bioactive metabolites have been isolated and characterized from actinomycetes, leading to the development of genetic engineering approaches to develop new bioactive compounds (Olano *et al*., 2011). In the last decade, the increasing efficiency and reduced cost of DNA sequencing has prompted researchers to join whole prokaryotic genome-sequencing projects (Lasken, 2012; Loman *et al*., 2012). At the present time, at least 5611 bacterial genomes are available, 779 corresponding to actinobacterias and among them 214 to *Streptomyces* species (http://www.ncbi.nlm.nih.gov/genomes/MICROBES/microbial_taxtree.html). This, together with the improvement of bioinformatics annotation (Torrieri *et al*., 2012; Wood *et al*., 2012) and biosynthesis gene clusters search tools (Fedorova *et al*., 2012) has shown that the metabolic capabilities of microorganisms known to produce bioactive compounds have been clearly underestimated (Nett *et al*., 2009). Some of the first sequenced actinomycete genomes corresponded to *Streptomyces avermitilis*, *Streptomyces coelicolor*, *Saccharopolyspora erythraea*, *Salinispora tropica* or *S. griseus*, producers of avermectin, actinorhodin, erythromycin, salinosporamide and streptomycin respectively (Nett *et al*., 2009). Analysis of these genome sequences has revealed the presence of numerous biosynthetic gene clusters (18 to 37) that might be involved in the biosynthesis of additional secondary metabolites belonging to different structural classes (Nett *et al*., 2009). These gene clusters are defined as cryptic or orphan because the identity of the natural product of the encoded pathway is unknown. The putative structure of these metabolites can be predicted, in some cases, using bioinformatics tools that take into consideration the function of the genes present in the cluster (Ziemert and Jensen, 2012). Various approaches have been described through genome mining to confirm the function of these pathways and to identify the products, including the activation of low-or non-expressed pathways, known as silent pathways (Zerikly and Challis, 2009; Winter *et al*., 2011).

*Streptomyces albus* J1074 is a derivative of *S. albus* G, defective in both restriction and modification enzymes of the SalI system (Chater and Wilde, 1976). Under normal growth conditions, this strain is not known to produce any bioactive natural product and it is widely used as a host for expression of *Streptomyces* secondary metabolite gene clusters (Baltz, 2010). In this work, we describe the identification of several compounds produced by *S. albus* J1074 using genome-mining approaches and activation of the expression of the corresponding gene clusters.

## Results

### Bioinformatic analysis of *S. albus* J1074 genome and identification of secondary metabolite gene clusters

The *S. albus* J1074 genome was sequenced and annotated by the Broad Institute (Cambridge, MA, USA), the sequence being available since 2008. It contains 6 823 670 bp [73.2% guanine-cytosine (GC) content] of which supercont3.1 genomic scaffold shotgun sequence represents 6 813 830 bp (accession number NZ_DS999645.1). This sequence comprises 5968 genes coding for 5902 predicted proteins. At the time the genome sequence was released, no production of secondary metabolites had been reported in *S. albus* J1074. However, *S. albus* G, parental strain of *S. albus* J1074 (Chater and Wilde, 1976), was shown (after treatment with N-methyl-N′-nitro-N-nitrosoguanidine and isolation of mutant J1670) to produce paulomycins A and B (Majer and Chater, 1987) and other *S. albus* strains were shown to produce the polyether salinomycin (Izumikawa *et al*., 2003a; Knirschová *et al*., 2007; Jiang *et al*., 2012; Yurkovich *et al*., 2012). We searched the genome sequence of *S. albus* J1074 chromosome using the bioinformatic tool antibiotics and Secondary Metabolite Analysis Shell (antismash) (Medema *et al*., 2011; Blin *et al*., 2013), and we found 26 secondary metabolite biosynthesis gene clusters predicted (Table 1). One additional cluster (cluster 23, Table 1), containing genes directing the biosynthesis and attachment of deoxyhexoses, was not identified by antismash but rather by specific search in the *S. albus* J1074 chromosome for genes encoding Nucleotide DiPhosphate-stereochemistry of sugar (NDP-D)-glucose synthase and NDP-D-glucose 4,6-dehydratase, both required for the biosynthesis of 6-deoxyhexoses.

**Table 1:** Secondary metabolite biosynthesis gene clusters identified in *S. albus* J1074 by genome mining

Cluster	Location^a^	Type	Predicted product^a^
1	SSHG_00001–00036	Type I PKS-NRPS	Unknown
2	SSHG_00040–00050	Lantipeptide	Class II lantibiotic
3	SSHG_00051–00053	Type I PKS-NRPS	Unknown
4	SSHG_00054–00069	Type I PKS-NRPS	Antimycins
5	SSHG_00076–00101	Type I PKS	Heptaene macrolide
6	SSHG_00144–00145	Type III PKS	THN, flaviolin
7	SSHG_00244–00268	Terpene	Isorenieratene
8	SSHG_00271	Bacteriocin	Linocin M18-family
9	SSHG_00311–00315	NRPS	Blue pigment
10	SSHG_00980–00990	Ectoine synthase	5-Hydroxyectoine
11	SSHG_01746–01756	Siderophore	Desferrioxamine
12	SSHG_01964–02004	NRPS	Unknown
13	SSHG_02468–02510	NRPS	Lipopeptide
14	SSHG_02783–02815	NRPS	Unknown
15	SSHG_03580–03598	Lantipeptide	Class III lantibiotic
16	SSHG_03731	Bacteriocin	Lactococcin 972-family
17	SSHG_03888–03912	Lantipeptide	Class I lantibiotic
18	SSHG_04343–04344	Terpene	Albaflavenone
19	SSHG_04630	Terpene	Geosmin
20	SSHG_04857–04868	Siderophore	Unknown
21	SSHG_04926–04963	NRPS	Lipopeptide
22	SSHG_05166–05175	Bacteriocin	Unknown
23^b^	SSHG_05313–5354	Deoxysugars	Glycosylated compound
24	SSHG_05575	Bacteriocin	Unknown
25	SSHG_05647–05659	Terpene	Hopene
26	SSHG_05699–05729	Type I PKS-NRPS	Polycyclic tetramate macrolactam
27	SSHG_05882–05890	NRPS	Unknown

a Location and predicted products based on antismash analysis of *S. albus* J1074 genome sequence (accession number NZ_DS999645.1) and individual blast analysis of selected genes.

b Cluster 23 was not located by antismash analysis but only by Blast analysis of individual genes.

Twelve clusters containing modular enzyme-coding genes [polyketide synthase (PKS) or non-ribosomal peptide synthetase (NRPS)] have been identified in *S. albus* J1074. Two of them (clusters 5 and 6) contain PKS genes belonging to type I and type III PKS respectively. The first one (cluster 5) could correspond to a type I PKS involved in the biosynthesis of a heptaene macrolide of the polyene family because of its high degree of similarity with the FR-008/candicidin cluster identified in several streptomycetes such as *S. griseus* IMRU 3570, *Streptomyces* sp. FR-008 or *Streptomyces* sp. S4 (Campelo and Gil, 2002; Chen *et al*., 2003; Seipke *et al*., 2011). The other PKS cluster (cluster 6) contains a type III PKS possibly involved in the production of 1,3,6,8-tetrahydroxynaphthalene and its auto-oxidation product flaviolin (Izumikawa *et al*., 2003b) because this cluster is well conserved in several actinomycetes, particularly *S. coelicolor* A3(2) and *S. avermitilis* MA-4680 (Nett *et al*., 2009; Craney *et al*., 2013; Ikeda *et al*., 2013). In addition, four clusters (clusters 1, 3, 4 and 26) contain hybrid type I PKS-NRPS genes. In the case of clusters 1 and 3, despite the fact that most of the amino acids incorporated by each adenylation domain can be predicted by several bioinformatic tools (Weber *et al*., 2009), it is quite hazardous to propose even a presumptive structure for the product of these clusters. On the other hand, cluster 4 and 26 might be involved in the biosynthesis of antifungal compounds. Cluster 4 is conserved among different *Streptomyces* in particular in *Streptomyces* sp. S4 (Seipke *et al*., 2011) and cluster 26 shows high similarity to *Lysobacter enzymogenes* strain C3 cluster involved in the production of a heat-stable antifungal factor (Yu *et al*., 2007). Six additional clusters are comprised of NRPS genes, five of them not conserved in other actinomycetes (clusters 12, 13, 14, 21 and 27). The NRPSs of two of them show high similarity to lipopeptide biosynthesis gene clusters (clusters 13 and 21), but for the same reasons mentioned above, no presumptive structure can be proposed. In addition, the NRPS-encoding gene *sshg_00313* from cluster 9 shows a high degree of similarity to genes involved in the biosynthesis of a blue pigment produced by different bacteria such as *Erwinia chrysanthemi* or several *Streptomyces* species (Reverchon *et al*., 2002; Takahashi *et al*., 2007; Novakova *et al*., 2010; Yu *et al*., 2013).

There are ten additional clusters involved in the biosynthesis of other peptides or amino acidic compounds such as siderophores (clusters 11 and 20), ribosomally derived lantipeptides (clusters 2, 15 and 17), bacteriocins (clusters 8, 16, 22 and 24) belonging to different families and ectoine (cluster 10, Table 1). Cluster 11 might be involved in the biosynthesis of desferrioxamine, a cluster well conserved among different streptomycetes (Barona-Gómez *et al*., 2004; 2006; Nett *et al*., 2009). Cluster 10, present in most actinomycetes (Nett *et al*., 2009), might determine the biosynthesis of osmoprotectant ectoine. The production of ectoine and 5-hydroxyectoine have been shown in *S. coelicolor* A3(2) to be triggered upon exposure to high salinity or elevated temperature (Bursy *et al*., 2008). Four clusters identified by antiSMASH determine the production of known terpenes (clusters 7, 18, 19 and 25) all of them also produced by both *S. coelicolor* A3(2) and *S. avermitilis* MA-4680 (Craney *et al*., 2013; Ikeda *et al*., 2013). Cluster 23 is the only one from *S. albus* J1074 identified by the presence of genes determining the biosynthesis of 2,6-deoxyhexoses. This cluster contains in addition two genes encoding glycosyltransferases (*sshg_05324* and *sshg_05335*).

### Activation of a small NRPS cluster for the biosynthesis of the blue pigment indigoidine

**Figure 1:**
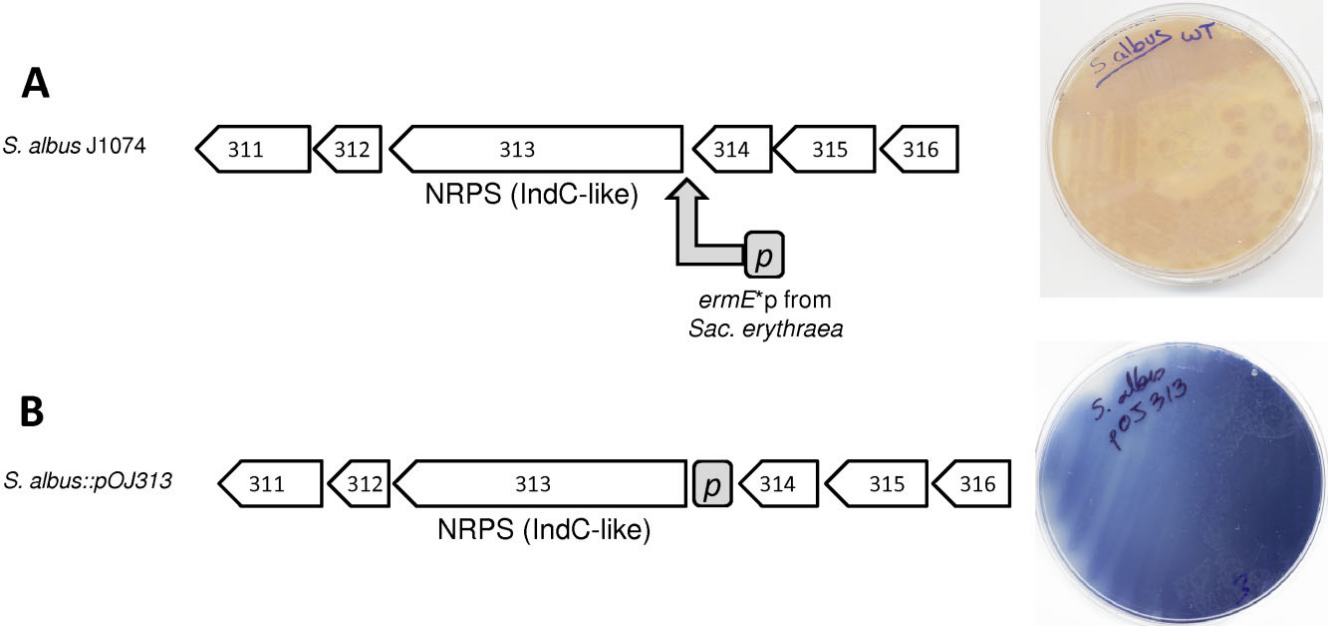
A. Genetic organization of cluster 9 and *S. albus* J1074 phenotype when grown in R5A solid medium. The grey arrow shows the location where the ermE*p was introduced using pOJ313. B. Genetic organization of activated cluster 9 and *S. albus::pOJ313* phenotype when grown in R5A solid medium.

In order to activate some cryptic/silent gene clusters in *S. albus* J1074, we decided to use a strategy based on the insertion of a strong and constitutive promoter in front of a selected gene of the cluster. We used the promoter of the erythromycin resistance gene (*erm*E*p) of *Sac. erythraea* (erythromycin producer). As a proof of concept, we decided to apply this strategy to a small NRPS cluster. In cluster 9, the *sshg_00313* gene codes for a single module NRPS that resembles the *bpsA* genes from *S. lavendulae* (Takahashi *et al*., 2007) and from *S. aureofaciens* (Novakova *et al*., 2010) and the *indC* genes from *S. chromofuscus* (Yu *et al*., 2013) and from *Erwinia chrysanthemi* (Reverchon *et al*., 2002). Analysis of the amino acid sequence of this gene indicates that would code for a NRPS containing an oxidation domain that is embedded in an adenylation domain with the signature sequence DAWQFGLINK for recognition of L-glutamine. In addition, SSHG_00313 contains a thiolation and thioesterase domains, structural organization shared with the BpsA and IndC homologues previously mentioned. Surrounding the *sshg_00313*, there are other genes for which homologues are also found in the vicinity of *bpsA* and *indC* in *Erwinia* and *Streptomyces* species such as: SSHG_00311 IndA-like, SSHG_00314 IndB-like, transmembrane transporter SSHG_00315 and phosphoribosyl transferase SSHG_00316. On the other hand, SSHG_00312 encodes a 4-oxalocrotonate tautomerase that is fused to *S. chromofuscus* IndC NRPS. The *indC*-like *bps*A gene is involved in the biosynthesis of the blue pigment indigoidine by condensation of two L-glutamines, this being the only activity required to generate the blue pigment (Müller *et al*., 2012). Under normal laboratory cultivation conditions, cultures of *S. albus* J1074 on agar plates do not show any blue pigmentation at all (Fig. 1A). Therefore, we considered that cluster 9 of *S. albus* J1074 might also be involved in the biosynthesis of this pigment, its expression being silent under normal cultivation conditions. To activate this small cluster, we inserted the *erm*E*p in front of the *sshg_00313* gene through homologous recombination using plasmid pOJ313 (Fig. 1A), and the result was that the resulting recombinant strain produced a blue colour (Fig. 1B), indicating that expression of the cluster was activated.

### Activation and identification of a cluster for the hybrid PK-NRP 6-*epi*-alteramides A and B

**Figure 2:**
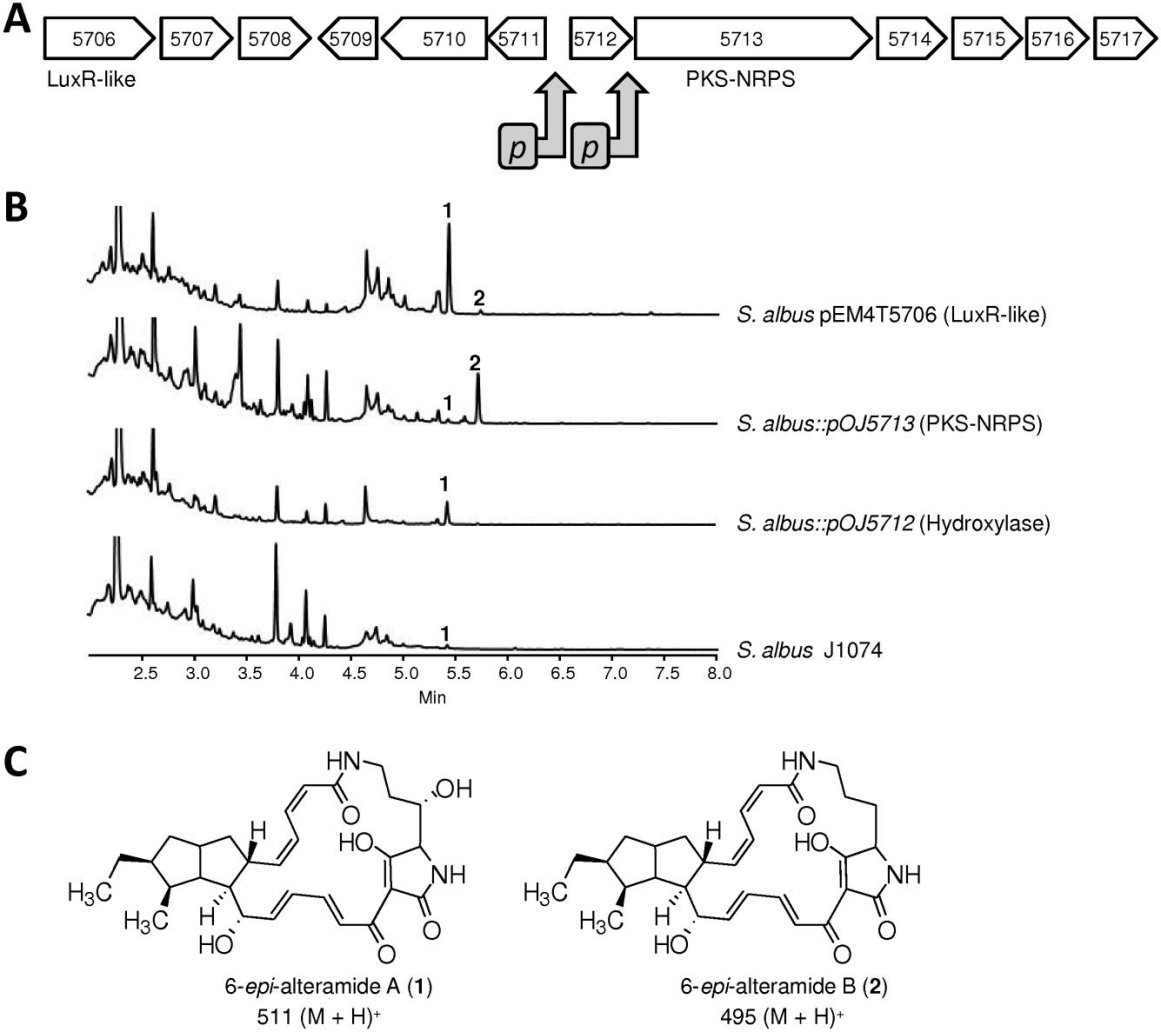
A. Genetic organization of *S. albus* J1074 cluster 26. The grey arrows show the location where the ermE*p was introduced using pOJ5712 and pOJ5713. B. UPLC chromatograms, monitored at 272 nm, of *S. albus* J1074, *S. albus:*:*pOJ5712*, *S. albus::pOJ5713* and *S. albus* pEM4T5706 grown during 7 days in R5A liquid medium. C. Chemical structure of novel compounds 6-*epi*-alteramide A (1) and 6-*epi*-alteramide B (2).

One of the clusters in *S. albus* (cluster 26) contains a gene coding for a hybrid type I PKS-NRPS (*sshg_05713*). This protein contains nine domains organized in two modules, one PKS module containing ketosynthase, acyltransferase, dehydratase, ketoreductase and acyl carrier domains, and an NRPS module composed by condensation, adenylation, peptidyl carrier protein and thioesterase domains. The PKS acyltransferase might incorporate malonate because it contains the characteristic GHSxGE signature while the NRPS adenylation domain would incorporate ornithine based in its conserved motif and characteristic-binding pocket signature DVGEIGSIDK. Surrounding the hybrid PKS-NRPS gene, there are several other genes coding for enzymatic activities possibly participating in the biosynthesis of a hybrid polyketide-peptide compound (cyclase/dehydrase SSHG_05711; flavin adenine dinucleotide-dependent oxidoreductases SSHG_05714 and SSHG_05715; oxidoreductase SSHG_05716; cytochrome P450 SSHG_05717 and hydroxylase SSHG_05712). This cluster showed similarity with other clusters from *Streptomyces* sp. SPB74 and *Streptomyces* sp. SPB78 that have been shown to be involved in the biosynthesis of the polycyclic tetramate macrolactams frontalamides A and B (Blodgett *et al*., 2010). To identify the compound synthesized by this cluster in *S. albus*, we inserted independently the ermE*p in front of *sshg_05712* and of *sshg_05713* using pOJ5712 and pOJ5713 respectively (Fig. 2A). Analysis by high-performance liquid chromatography-mass spectrometry (HPLC-MS) of ethyl acetate extracts of the corresponding recombinant strains showed the appearance in the case of the *sshg_05712* gene of a peak with a mass of *m/z* 511 [M + H]^+^, and in the case of the *sshg_05713* gene, the same peak was observed and an additional peak with a mass of *m/z* 495 [M + H]^+^ was also present (Fig. 2B). Mass analysis allowed us to discard the possibility that they were coincident with frontalamides A and B because these compounds have a different mass [*m/z* 525 (M + H)^+^ and *m/z* 509 (M + H)^+^, respectively] and slightly different absorption spectrum, and also to exclude other members of the family such as ikarugamycin [mass *m/z* 481 (M + H)^+^], cylindramide [mass *m/z* 467 (M + H)^+^] or dihydromaltophilin [mass *m/z* 513 (M + H)^+^]. The first peak was detected as a very tiny peak in extracts of the wild-type strain and the second one was undetected. The two compounds were isolated, purified and their structures elucidated by nuclear magnetic resonance (NMR) and mass spectrometry (MS; see Supporting information) and found to correspond to novel compounds 6-*epi*-alteramide A and 6-*epi*-alteramide B (Fig. 2C). They differ in the presence of a hydroxyl group at C26 of 6-*epi*-alteramide A, which is absent in 6-*epi*-alteramide B.

In the vicinity of *sshg_05712*, 7.6 kb upstream, we found *sshg_05706* that would code for a transcriptional regulator of the LuxR-family. The region comprising from *sshg_05706* to *sshg_05711* is not conserved in other clusters involved in the biosynthesis of polycyclic tetramate macrolactams (Blodgett *et al*., 2010). However, to assess the possible involvement of SSHG_05706 in the regulation of the biosynthesis of 6-*epi*-alteramides, we expressed *sshg_05706* under the control of ermE*p using a high copy number plasmid (pEM4T5706). *Streptomyces albus* J1704 expressing *sshg_05706* overproduced 6-*epi*-alteramide A and small amounts of 6-*epi*-alteramide B were also detected (Fig. 2B).

### Simultaneous activation of candicidins and antimycins gene clusters

**Figure 3:**
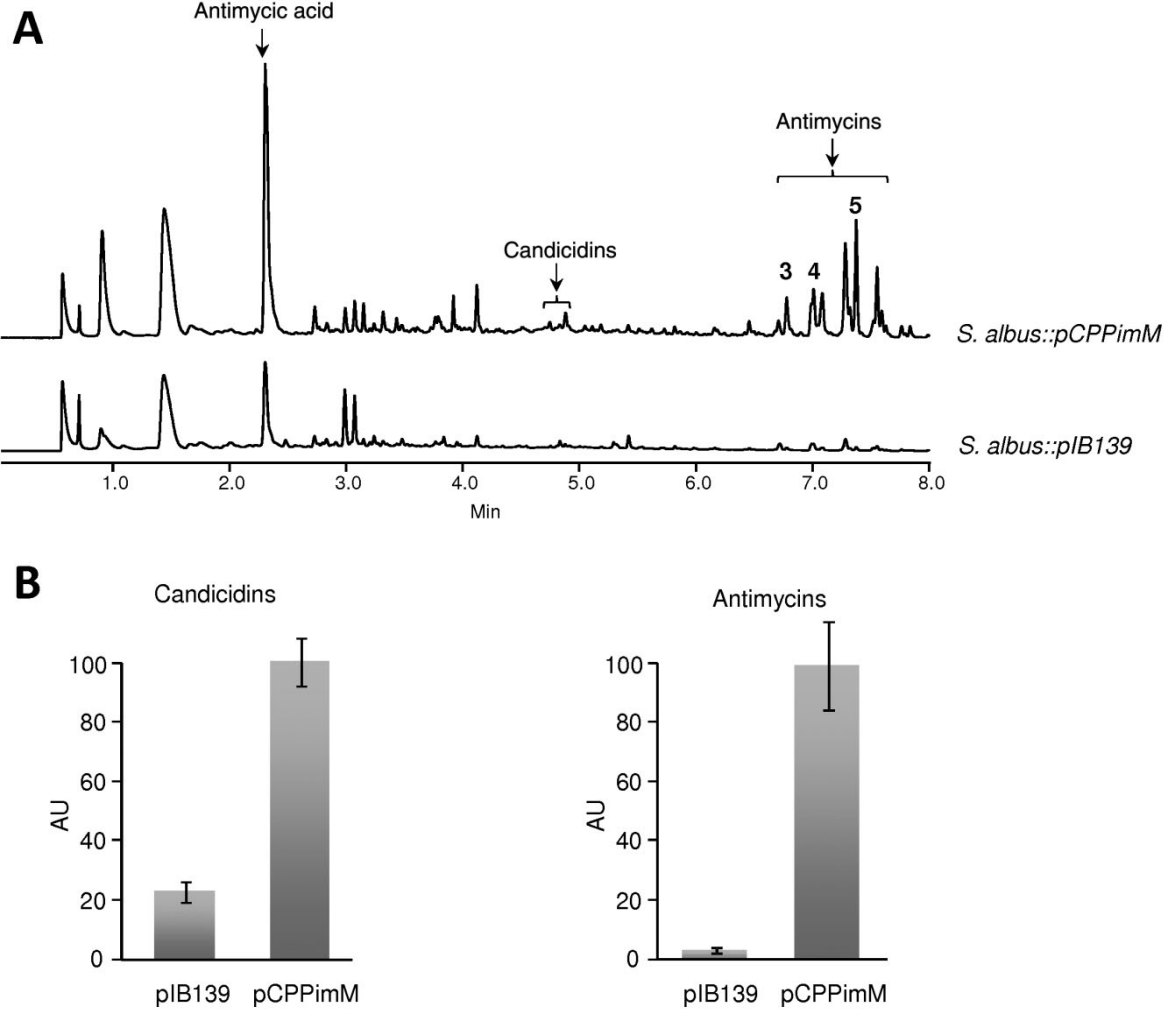
A. UPLC chromatograms, monitored at 244 nm, of *S. albus::pCPPimM* expressing regulatory gene pimM cloned in pIB139 and *S. albus*::*pIB139* used as a control, both grown during 7 days in R5A liquid medium. The arrows indicate the mobility of candicidins and antimycins used as standards. B. Production of candicidins and antimycins by *S. albus* J1074 [wild-type (WT)], *S. albus:*:*pIB139* (pIB139) and *S. albus:*:*pCPPimM* (pCPPimM) grown during 7 days in R5A liquid medium.

**Figure 4:**
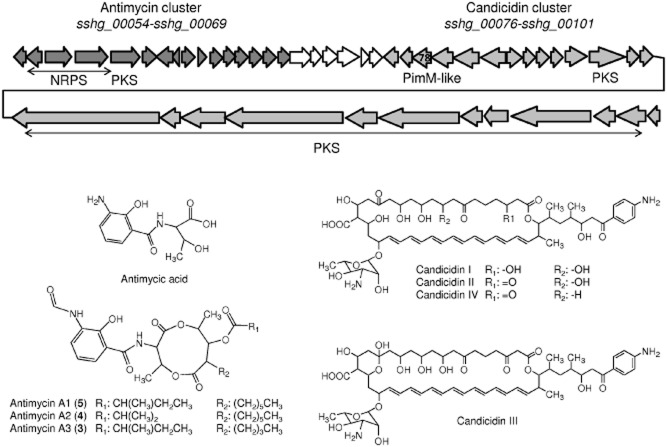
Genetic organization of *S. albus* J1074 clusters 4 and 5 involved in the biosynthesis of antimycins and candicidins, respectively, and chemical structure of compounds of these families.

One of the largest clusters in *S. albus* J1074 genome is that of a type I polyketide (*sshg_00076* to *sshg_00101*) that could code for a heptaene macrolide. It comprises a region of approximately 137 kb, and it contains a large number of type I PKS-coding genes. Most of the genes of this cluster shows high similarity with genes involved in the biosynthesis of the macrolide polyene FR-008/candicidin (Campelo and Gil, 2002; Chen *et al*., 2003; Seipke *et al*., 2011). Polyenes possess a characteristic absorption spectrum with maxima at 230, 360, 380 and 400 nm. However, under different incubation conditions, no clear peaks were detected in extracts of *S. albus* that could indicate the presence of a polyene. To attempt the activation of this cluster and to identify the compound it codes, we used a regulatory gene from another cluster in another organism. This was the *pimM* gene from the pimaricin cluster in *S. natalensis* that codes for a positive activator of the LuxR-family in the biosynthesis of pimaricin (Antón *et al*., 2007). This gene was expressed in *S. albus* using an integrative vector under the control of the ermE*p promoter (pCPPimM). Upon integration of the vector into the *S. albus* chromosome, we detected the appearance of small peaks with the characteristic absorption spectra of heptaenes (Fig. 3A). These peaks, although small, were reproducible and clearly showed a significant increase in the production yields (fourfold) (Fig. 3B). Mass analysis showed ions of *m/z* 1111, 1109, 1109 and 1093 [M + H]^+^ and co-migration with pure candicidins I, II, III and IV respectively, used as standard (Fig. 4). Interestingly, we also observed several peaks in the chromatogram with a later chromatographic retention and another peak with an earlier retention time (Fig. 3A). Mass analyses of these peaks showed ions of *m/z* 255, 549, 535 and 521 [M + H]^+^ respectively, and absorption spectra with maxima at 229 and 319 nm. Co-migration with pure samples used as standards revealed that they corresponded to different members of the antimycins family (antimycins A1, A2 and A3) and its biosynthetic precursor antimycic acid (Fig. 4). Antimycins showed a magnified production yield upon expression of PimM (29-fold) (Fig. 3B). A careful reanalysis of the *S. albus* genome prompted us to identify one of the PKS-NRPS clusters located in the vicinity of the candicidin cluster (genes *sshg_00054* to *sshg_00069*) as the putative antimycin cluster (Fig. 4). This cluster showed similarity with other antimycin clusters described from *Streptomyces* S4, *Streptomyces ambofaciens* and other *Streptomyces* strains (Seipke *et al*., 2011; Yan *et al*., 2012; Aigle *et al*., 2013). In the *S. albus* candicidin cluster, there is a *pimM* homologous gene (*sshg_00078*). We also expressed this gene (pIB00078) in *S. albus* using the same integrative vector and we obtained similar results as with PimM (data not shown).

### Identification of the paulomycin gene cluster

**Figure 5:**
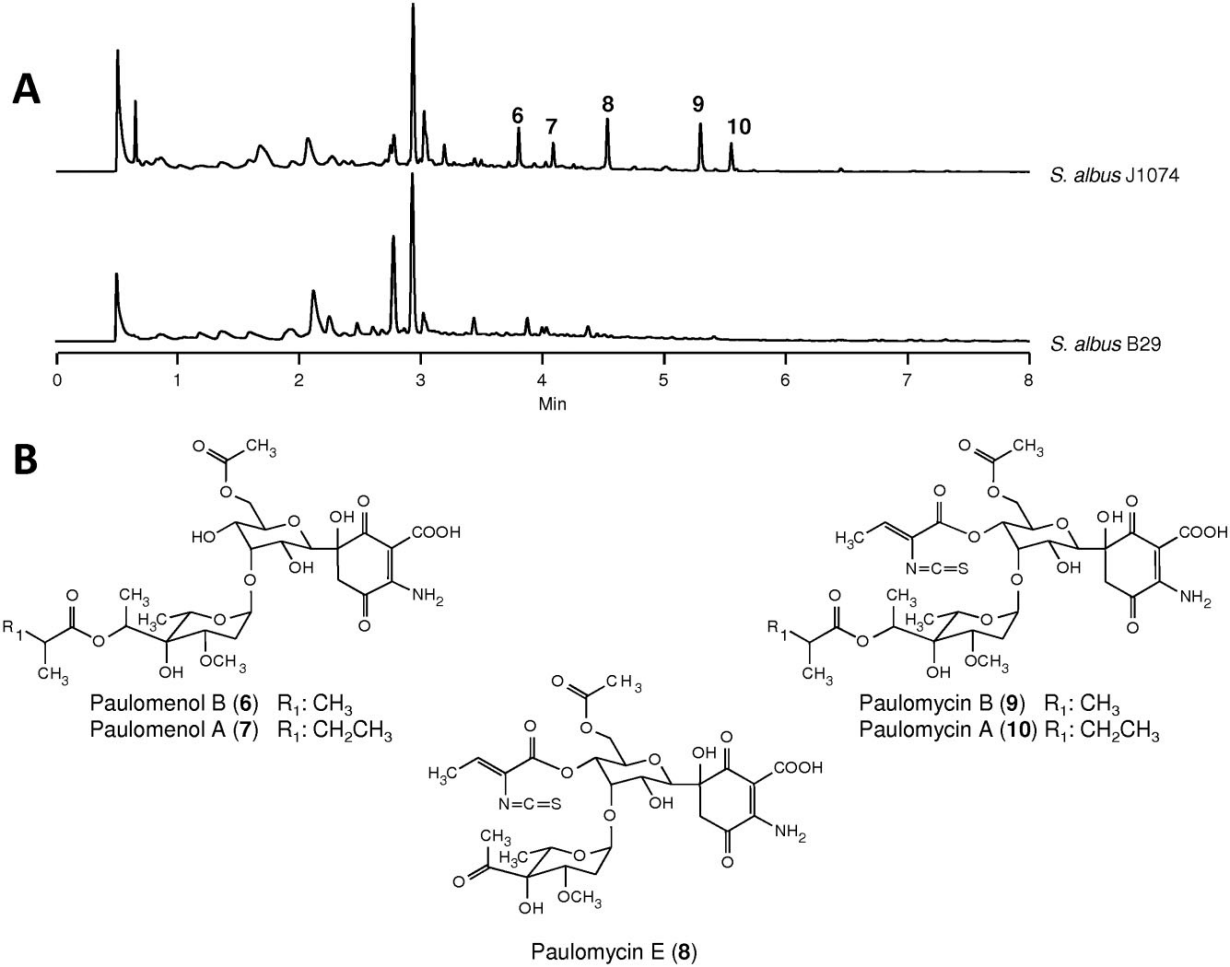
A. UPLC chromatograms, monitored at 244 nm, of *S. albus* J1074 and mutant *S. albus* B29 in which *sshg_05727* and *sshg_05728* have been deleted. Peaks absent in the mutant strain are labelled as compounds 6 to 10. B. Chemical structures of paulomenols B (6) and A (7) and paulomycins E (8), B (9) and A (10).

**Figure 6:**
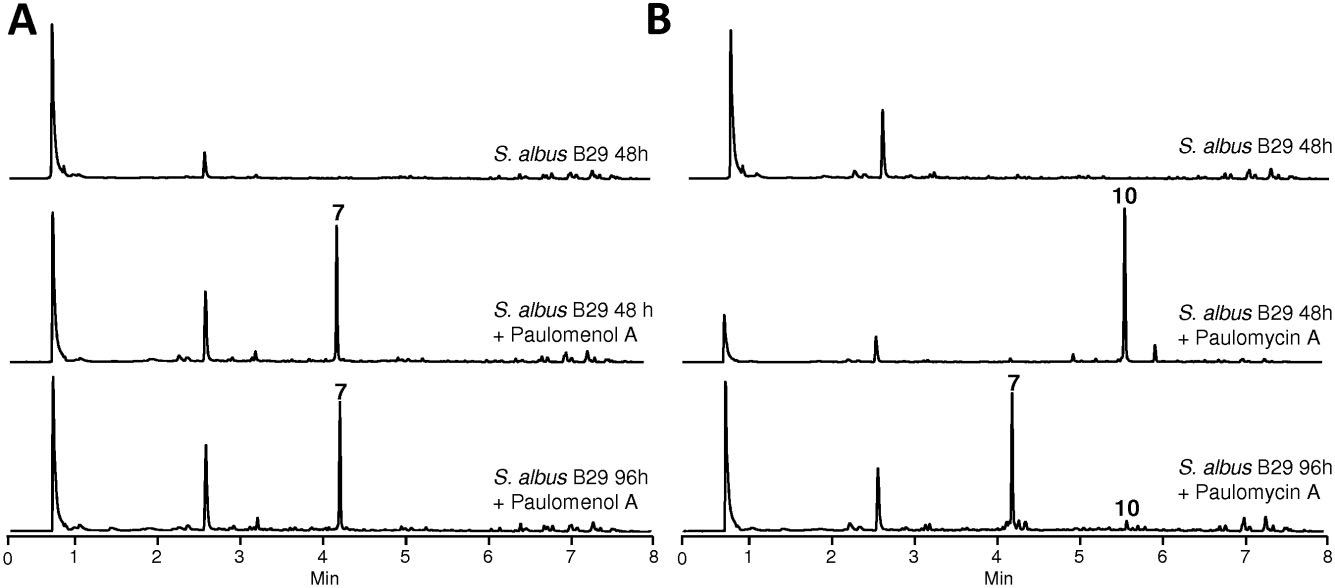
A. UPLC chromatograms, monitored at 244 nm, of mutant *S. albus* B29 grown in R5A liquid medium and fed with 50 μg ml^−1^ of paulomenol A. B. UPLC chromatograms, monitored at 244 nm, of mutant *S. albus* B29 grown in R5A liquid medium and fed with 50 μg ml^−1^ of paulomycin A.

Analysis of the genome sequence of *S. albus* J1074 for the presence of gene clusters coding for glycosylated secondary metabolites only revealed the presence of one cluster containing genes involved in the biosynthesis of 6-deoxyhexoses (cluster 23). This cluster would comprise a region of approximately 60 kb and, in addition to several genes involved in 6-deoxysugar biosynthesis, two glycosyltransferases and three putative acyltransferases, we also observed the presence of genes coding for enzymes acting on chorismate to generate 3-hydroxyanthranilic acid or other chorismate-derived compounds. We therefore anticipated that the compound coded by this cluster could contain a central core derived from chorismate, which would be glycosylated with two deoxysugars and it should contain three acylations. To identify the compound produced by this cluster, we generated a mutant (*S. albus* B29) by the simultaneous deletion of two consecutive genes involved in chorismate modification, *sshg_05327* and *sshg_05328* that would code for an anthranilate synthase and an isochorismatase respectively. Inactivation of these genes produced the disappearance of five peaks from the chromatogram (Fig. 5A). Two of them (peaks 6 and 7) shared a similar absorption spectrum with maxima at 244 and 321 nm and mass analysis revealed ions of *m/z* 648 and 662 [M + H]^+^ respectively. The other three peaks (peaks 8, 9 and 10) shared a different spectrum with maxima at 234, 275 and 323 nm and masses of *m/z* 701, 773 and 787 [M + H]^+^ respectively. At this stage, it is important to mention that the presence of these five peaks in ethyl acetate extracts of *S. albus* J1074 was very variable and we could not find a reproducible pattern for production of these compounds using different batches of media or changing the culture media. The five compounds were purified and found to correspond to paulomycin A (peak 10), paulomycin B (peak 9), paulomycin E (peak 8), paulomenol A (peak 7) and paulomenol B (peak 6) by comparing their chromatographic mobilities, absorption spectra, masses and co-migration with authentic samples of these compounds (Braña *et al*., 2014). These compounds were originally isolated from *S. paulus* (Argoudelis *et al*., 1982; 1988a,b). The main difference between the two groups of compounds is the presence in the paulomycins of an acylation with a paulic acid residue containing an isothiocyanate residue that is not present in paulomenols and that is responsible of the absorbance peak at 275 nm observed in paulomycins (Argoudelis *et al*., 1988b). Paulomenols have been proposed to be true intermediates in the biosynthesis of paulomycins (Argoudelis *et al*., 1988b). However, some experiments prompted us to speculate that perhaps paulomenols were not real intermediates but rather degradation products of paulomycins. To answer this question, we carried out feeding experiments using as biotransformation host the non-producing deletion mutant described before (*S. albus* B29) blocked in early steps of the biosynthesis of paulomycins (Fig. 6). We fed paulomenol A to this mutant after 48 h of growth, and after a further 48 h, we could not observe any conversion of paulomenols into paulomycins (Fig. 6A). In contrast, when we fed the same mutant with paulomycin A, there was a progressive appearance of paulomenol A in parallel to a decrease in paulomycin A (Fig. 6B). These experiments demonstrated that paulomycin A was quite unstable and paulomenols arise as degradation products upon incubation. Therefore, the conclusion is that paulomenols are not real biosynthetic intermediates in the biosynthesis of paulomycins but rather degradation products.

## Discussion

Filamentous bacteria of the actinomycetes family are amazingly prolific in the number of bioactive they can produce. About 75% of the antibiotics are produced by actinomycetes and most of them are synthesized by the genus *Streptomyces* (Demain, 2013). Nevertheless, in the last decade, the number of novel chemical entities from actinomycetes has declined concomitantly with a decrease of new drug discovery by the pharmaceutical industry. However, the development of whole genome-sequencing technology and its application to actinomycetes has revealed that these microorganisms have a greater potential for the production of bioactive natural products that was anticipated (Challis, 2008; Corre and Challis, 2009; Nett *et al*., 2009). Many *Streptomyces* species, previously known to produce one or two bioactive compounds, have been shown to possess gene clusters for a larger number of secondary metabolites. However, most of them are not expressed during standard fermentations (Olano *et al*., 2008; Baltz, 2011). Therefore, there is a big potential in actinomycetes for the discovery of novel bioactive natural products that can be useful for clinical or pharmaceutical applications. A key issue for the success in finding novel compounds is to find strategies to turn on (or turn up) the expression of these cryptic or low (or non) expressed biosynthesis pathways to identify the corresponding compounds and to test their biological activities. We have applied three different strategies for the activation and identification of silent/cryptic biosynthesis pathways in *S. albus* J1074: insertion of a strong promoter, overexpression of a positive regulatory gene and generation of a non-producing mutant. *Streptomyces albus* J1704 is a *Streptomyces* strain not known for its capability to produce a number of bioactive compounds but widely used by researchers in the actinomycetes field as a host for gene expression. Bioinformatic analysis of the genome of *S. albus* J1074 showed that in agreement with what has been found in other actinomycetes, it possesses a large number of gene clusters for secondary metabolites. Assuming that the lack of expression of some gene clusters could be due to the absence of some signals to be recognized by specific promoter regions of the cluster, we selected as one of the strategies the insertion of a strong and constitutive promoter in front of some key genes of selected clusters. This was the promoter of the erythromycin resistance genes, ermE*p. The proof of concept indicating that this strategy could function was successfully obtained through the activation of the expression of a small cluster coding for the blue pigment indigoidine. This strategy was also used to activate the expression of a cluster coding for a hybrid PK-NRP compound of the polycyclic tetramate macrolactams family. Insertion of the ermE*p in front of two genes of the cluster induced the biosynthesis of two novel compounds of this family, 6-*epi*-alteramides A and B, epimers at position 6 of alteramides A and B, compounds isolated from the bacterium *Alteromonas* sp. living in symbiosis with a marine sponge (Shigemori *et al*., 1992). This approach bypasses the natural induction of the expression of the cluster by the corresponding intracellular signal.

A second strategy we have used for cluster activation was the overexpression of positive regulatory genes. This is not a novel strategy for yield enhancement and has been used in a considerable number of systems (Bibb, 2005; Olano *et al*., 2008). Such approach can use regulatory genes from the same cluster or heterologously expressed regulatory genes from other clusters. We have used both approaches in two different clusters. The 6-*epi*-alteramides gene cluster was also activated by the overexpression in *S. albus* of a regulatory gene (*sshg_05706*) of the same organism belonging to the LuxR-family and located 7.6 kb upstream of the hybrid PKS-NRPS gene. Gene clusters for the biosynthesis of different members of this macrolactam family have been shown to be present in different actinomycetes (Blodgett *et al*., 2010). However, when we searched for the presence of homologous genes to *sshg_05706* in several of these organisms, we could not find any homologous gene neither in the same position nor in the vicinity of these clusters. At present, it is not clear if this LuxR-like gene of *S. albus* is a pathway-specific regulator or a pleiotropic regulatory gene positively affecting the expression of 6-*epi*-alteramides among other functions. However, the high similarity among these clusters both at the gene organization level and at the DNA and protein sequence level and the absence of LuxR-like genes in these organisms are more in favour to support the view that the LuxR-like SSHG_05706 of *S. albus* is a pleiotropic regulator. Expression of a LuxR-like gene from *S. ambofaciens* has been also used to trigger the expression of stambomycins A-D, 51-membered glycosylated macrolides (Laureti *et al*., 2011).

Activation of clusters by the heterologous expression of a regulatory gene gave us surprising results. From the bioinformatic analysis, we suspected the presence of a cluster coding for the polyene candicidins. PimM is a regulatory gene of the pimaricin pathway in *S. natalensis* that combines an N-terminal PAS sensory domain with a C-terminal helix–turn–helix motif of the LuxR type for DNA binding (Santos-Aberturas *et al*., 2010). Insertion of this gene in the chromosome of *S. albus* allowed the identification of candicidins as products of the corresponding gene cluster. Surprisingly, a cluster for the biosynthesis of antimycins was also activated, this cluster being located in the vicinity of the candicidins cluster. The simultaneous activation of both clusters suggests that either the two of them use a common regulatory gene or that there could be some kind of cross-talk between them. Cross-regulation between different biosynthesis gene clusters has been reported in several *Streptomyces* (Fuente *et al*., 2002; Huang *et al*., 2005). It is interesting to mention that gene clusters for the biosynthesis of candicidins are quite frequent in the genomes of actinomycetes (Haeder *et al*., 2009; Jørgensen *et al*., 2009) and quite frequently an antimycins cluster is also present closely linked to the candicidins cluster (Seipke *et al*., 2011; Yan *et al*., 2012; this manuscript). Both compounds show antifungal activity, and it is possible that through evolution actinomycetes have preserved these biosynthesis pathways to combat or to protect themselves or associated organisms in which they live in symbiosis (i.e. ants) of fungal infections.

Glycosylated compounds are quite frequent within bioactive natural products (Weymouth-Wilson, 1997; Méndez and Salas, 2001; Salas and Méndez, 2007). However, searching in the genomes of actinomycetes reveals that the presence of gene clusters for glycosylated compounds is rare in this group of organisms. In the genome of *S. albus* only one cluster for a glycosylated compound is present, and it was not identified with antismash. We identified the compounds produced by this cluster using a third strategy, the generation of a non-producing mutant by deleting two key genes in the cluster. We cannot consider this cluster as silent/cryptic because the peaks corresponding to paulomycins and paulomenols were present in ethyl acetate extracts of the wild-type strain. However, regulation of the expression of this cluster seems to be very loose because there were clear qualitative and quantitative variations in the presence and levels of production of the five compounds. It is worth mentioning that two of these compounds, paulomenols A and B, were initially supposed to be intermediates in the biosynthesis of paulomycins (Argoudelis *et al*., 1988b). However, they were not converted into paulomycins when fed to a mutant blocked in early stages of the biosynthesis while feeding paulomycins gave rise to the appearance of paulomenols. This is a clear indication of the instability of paulomycins that could easily lose the paulic acid residue, thus generating paulomenols. According to this experimental evidence, paulomenols would not be real biosynthetic intermediates but rather degradation products.

In conclusion, we have demonstrated the presence in *S. albus* J1074 of gene clusters for the biosynthesis of two hybrid PK-NRP (antimycin and 6-*epi*-alteramides), a type I PK (candicidin), an NRP (indigoidine) and a glycosylated family of compounds (paulomycins). Activation of the expression of these clusters was achieved by insertion of a strong and constitutive promoter and through the overexpression of positive regulatory genes. In the case of paulomycins, the identification came from comparison of the HPLC profiles between the wild-type strain and a non-producing mutant. Several other clusters are also present in *S. albus*, and we are now proceeding using these and other strategies to attempt the identification of all bioactive compounds produced by this organism.

## Experimental procedures

### Strains, culture conditions and plasmids

Bacterial strains used in this work were *S. albus* J1074 (Chater and Wilde, 1976), *Escherichia coli* DH10B (Invitrogen, Grand Island, NY, USA) and ET12567 (pUB307) (Kieser *et al*., 2000) used for subcloning and intergeneric conjugation respectively. Growth medium for *S. albus* and mutants was tryptone soya broth; A medium was used for sporulation (Fernández *et al*., 1998) and R5A as production medium (Fernández *et al*., 1998). *Escherichia coli* media were those described in the literature (Sambrook *et al*., 1989). When plasmid-containing clones were grown, the medium was supplemented with the appropriate antibiotics: 100 μg ml^−1^ ampicillin, 20 μg ml^−1^ tobramycin, 25 μg ml^−1^ apramycin, 50 μg ml^−1^ thiostrepton, 10 μg ml^−1^ tetracycline, 25 μg ml^−1^ chloramphenicol or 50 μg ml^−1^ nalidixic acid.

Plasmids used in this work were pOJ260P (Olano *et al*., 2004) and pEFBAoriT, (Horna *et al*., 2011) for gene disruption and gene replacement respectively. pEM4T (Menéndez *et al*., 2006) and pIB139 (Wilkinson *et al*., 2002) were used for gene expression. pLHyg (Olano *et al*., 2004) was the source of the hygromycin resistance gene *hyg*. pCR-BLUNT (Invitrogen) was used for cloning polymerase chain reaction (PCR) products. pCPPimM (Antón *et al*., 2007) was used to expresses *pimM* under the control of ermE*p.

### DNA analysis of *S. albus* J1074 chromosome

Computer-aided database searching and sequence analysis were carried out using the bioinformatic tool antismash (Medema *et al*., 2011; Blin *et al*., 2013) and the blast program (Altschul *et al*., 1997). Analysis of PKS-and NRPS-predicted proteins were carried out using programs asmpks (Tae *et al*., 2007) and NRPS predictor (Rausch *et al*., 2005).

### DNA manipulation

Deoxyribonucleic acid manipulations were performed according to standard procedures for *E. coli* (Sambrook *et al*., 1989) and *Streptomyces* (Kieser *et al*., 2000). PCR conditions used for all amplifications were 97°C, 5 min.; 30 cycles of 95°C, 30 s, 50°C, 45 s and 68°C, 1 min and a final extension cycle at 68°C, 10 min. Pfx DNA polymerase (Invitrogen) and 2.5% dimethyl sulfoxide (DMSO) were used for all amplifications. To introduce the ermE*p promoter in front of the *sshg_00313* gene through homologous recombination, a 2 kb fragment upstream of *sshg_00313* containing its start codon was amplified using oligoprimers SA313A and SA313B (Supporting Information Table S1 in Supplementary Information). The corresponding PCR product was cloned in pCR-BLUNT (Invitrogen) and confirmed by DNA sequencing. Afterwards the fragment was subcloned into the BamHI-EcoRI-digested pOJ260P, where *sshg_00313* is located downstream of ermE*p promoter leading to plasmid pOJ313.

pOJ5712 and pOJ5713 were generated to clone the ermE*p promoter in front of the *sshg_05712* and *sshg_05713* genes through homologous recombination. A 1.53 kb PCR fragment containing the start codon of *sshg_05712* was amplified using oligoprimers SA5712A and SA5712B (Supporting Information Table S1). The PCR product was cloned in pCR-BLUNT and then subcloned into the PstI-XbaI-digested pOJ260P, leading to pOJ5712. pOJ5713 was generated using the same approach by amplification of a 1.58 kb PCR fragment, containing the start codon of *sshg_05712*, using oligoprimers SA5713A and SA5713B (Supporting Information Table S1).

For the expression of *ssh_05706* and *sshg_00078* (under the control of ermE*p) in *S. albus* J1074, the corresponding genes were amplified by PCR. The *sshg_05706* gene was amplified as a 2628 bp fragment using oligoprimers 11R1A1 and 11R1B1 (Supporting Information Table S1). Oligoprimers 078FW and 078RV (Supporting Information Table S1) were used to amplify an 800 bp fragment containing *sshg-00078*. After verification by sequencing of each PCR fragment, these were subcloned into the BamHI-EcoRI digested pEM4T (for *ssh_05706*) or pIB139 (for *sshg_00078*) leading to constructs pEM4T5796 and pIB00078 respectively.

Deletion of genes *sshg_05327* and *sshg_05328* was accomplished by amplification of two DNA fragments of 1.5 kb. Fragment A, amplified using the oligoprimers SA5238FW and SA5328RV (Supporting Information Table S1), was cloned into SpeI-SphI-digested pEFBAoriT leading to pT5328A. Fragment B, amplified using the oligoprimers SA5327FW and SA5327RV (Supporting Information Table S1), was cloned into NdeI-BamHI-digested pT5328A, leading to pT5328/27 where fragments A and B are flanking the apramycin resistance gene *aac(3)IV*. Finally, pT5328/27 was digested with XbaI, and the gene *hyg* from pLHyg was subcloned as a SpeI-NheI fragment to obtain plasmid p5328/27Hyg used for the generation of *S. albus* mutant B29.

### Analysis of metabolites by ultra performance liquid chromatography (UPLC) and liquid chromatography–mass spectrometry (LC-MS)

Whole cultures were extracted with ethyl acetate containing 1% formic acid (to enhance the extraction of compounds containing ionizing groups) and analysed by reversed phase chromatography in an Acquity UPLC instrument fitted with a BEH C18 column (1.7 μm, 2.1 × 100 mm; Waters), with acetonitrile and 0.1% trifluoroacetic acid (TFA) as solvents. Samples were eluted with 10% acetonitrile for 1 min, followed by a linear gradient from 10% to 100% acetonitrile over 7 min, at a flow rate of 0.5 ml min^−1^ and a column temperature of 35°C. For HPLC-MS analysis, an Alliance chromatographic system coupled to a ZQ4000 mass spectrometer and a SunFire C18 column (3.5 μm, 2.1 × 150 mm; Waters) was used. Solvents were the same as above and elution was performed with an initial isocratic hold with 10% acetonitrile during 4 min followed by a linear gradient from 10% to 88% acetonitrile over 26 min, at 0.25 ml min^−1^. MS analysis were done by electrospray ionization in the positive mode, with a capillary voltage of 3 kV and a cone voltage of 20 V. Detection and spectral characterization of peaks was performed in both cases by photodiode array detection in the range from 200 nm to 500 nm, using empower software (Waters) to extract bidimensional chromatograms at different wavelengths, depending on the spectral characteristics of the desired compound.

### Isolation of 6-*epi*-alteramides

Strains *S. albus::pOJ5712* and *S. albus::pOJ5713* were grown in R5A medium without sucrose for the purification of 6-*epi*-alteramide A (1) and B (2) respectively. In each case, 40 Erlenmeyer flasks (250 ml), each containing 50 ml of medium, were inoculated with spores and incubated in an orbital shaker (Climo-Shaker ISF4-X, Adolf Kühner AG, Basel, Switzerland) at 30°C and 250 r.p.m. during 3 days. The cultures were centrifuged, the supernatants were discarded and the pellets were extracted twice with ethyl acetate acidified with 1% formic acid. The organic extracts were evaporated in vacuo, and the resulting dry extract was re-dissolved in 3 ml of a mixture of DMSO and methanol (1:1). The compounds of interest were purified by preparative HPLC using a SunFire C18 column (10 μm, 10 × 250 mm; Waters). Compounds were chromatographed with mixtures of acetonitrile and 0.05% TFA in water in isocratic conditions optimized for each peak at 7 ml min^−1^. After every purification step, the collected compounds were diluted fourfold with water and were desalted and concentrated by solid-phase extraction (Sep-Pak C18, Waters). Finally, the compounds were dissolved in tert-butanol and lyophilized. The resulting yields were 2.7 mg of 6-*epi*-alteramide A (1) and 13.8 mg of 6-*epi*-alteramide B (2).

### Structural characterization of 6-*epi*-alteramides

6-*epi*-alteramide A (1) and B (2) were purified from cultures of *S. albus::pOJ5712* and of *S. albus::pOJ5713* respectively. Their structural elucidation was carried out using 1D ^1^H, 1D ^13^C, 2D ^1^H COSY, 2D ^1^H TOCSY, ^1^H, ^13^C heteronuclear multiple-quantum correlation-edited and heteronuclear multiple-bond correlation NMR experiments using deuterated methanol (CD_3_OD) as solvent (Supporting Information Figs. S1–S15 in Supplementary Information). NMR spectra were recorded at 24°C on a Bruker AVANCE III-600 MHz using a 1.7 mm microcryoprobe (Bruker). 6-*epi*-alteramide A and B NMR data are shown in Tables S2–S4.

## Supporting Information

**Fig. S1.** Key NOESY correlations observed in the structure of 6-*epi*-alteramide A. **Fig. S2.** ^1^H NMR spectrum of 6-*epi*-alteramide A (CD_3_OD, 500 MHz). **Fig. S3.** ^1^H NMR spectrum of 6-*epi*-alteramide A (1:1 CD_3_OD/CDCl3, 500 MHz). **Fig. S4.** COSY spectrum of 6-*epi*-alteramide A (1:1 CD_3_OD/CDCl_3_). **Fig. S5.** NOESY spectrum of 6-*epi*-alteramide A (1:1 vCD_3_OD/CDCl_3_, mixing time 600 msec). **Fig. S6.** HSQC spectrum of 6-*epi*-alteramide A (1:1 CD_3_OD/CDCl_3_). **Fig. S7.** HSQC spectrum of 6-*epi*-alteramide B (1:1 CD_3_OD/CDCl_3_). **Fig. S8.** ^1^H NMR spectrum of 6-*epi*-alteramide B (CD_3_OD, 500 MHz). **Fig. S9.** Comparison ^1^H NMR spectra of 6-*epi*-alteramide A (blue) and 6-*epi*-alteramide B (red) (1:1 CD_3_OD/CDCl_3_, 500 MHz). **Fig. S10.** COSY spectrum of 6-*epi*-alteramide B (CD_3_OD). **Fig. S11.** TOCSY spectrum of 6-*epi*-alteramide B (CD_3_OD, mixing time 90 msec). **Fig. S12.** NOESY spectrum of 6-*epi*-alteramide B (CD_3_OD, mixing time 600 msec). **Fig. S13.** HSQC spectrum of 6-*epi*-alteramide B (CD_3_OD). **Fig. S14.** Expansion (olefinic region) of the HSQC spectrum of 6-*epi*-alteramide B (CD_3_OD). Assignments are indicated by numbers. Circled signals correspond to the cycloaddition product. **Fig. S15.** Expansion (aliphatic region) of the HSQC spectrum of 6-*epi*-alteramide B (CD_3_OD). Assignments are indicated by numbers. **Table S1.** Primer sets used in this work for the amplification of DNA regions used in gene inactivation and gene expression. **Table S2.** 6-*epi*-alteramide A ^1^H and 13C NMR data acquired in 1:1 CD_3_OD/CDCl_3_ (500 MHz, 24 °C). **Table S3.** 6-*epi*-alteramide A ^1^H and 13C NMR data acquired in CD_3_OD (500 MHz, 24 °C). **Table S4.** 6-*epi*-alteramide B ^1^H and 13C NMR data acquired in CD_3_OD (500 MHz, 24 °C).
