# Supplementary material for: Activation and identification of five clusters for secondary metabolites in Streptomyces albus J1074
Source: Microb Biotechnol. 2014 Mar 4;7(3):242–56. doi: 10.1111/1751-7915.12116 (PMC3992020; doi:10.1111/1751-7915.12116)
Supplement: Supplementary file 1 — Fig. S1. Key NOESY correlations observed in the structure of 6-epi-alteramide A. Fig. S2. 1H NMR spectrum of 6-epi-alteramide A (CD3OD, 500 MHz). Fig. S3. 1H NMR spectrum of 6-epi-alteramide A (1:1 CD3OD/CDCl3, 500 MHz). Fig. S4. COSY spectrum of 6-epi-alteramide A (1:1 CD3OD/CDCl3). Fig. S5. NOESY spectrum of 6-epi-alteramide A (1:1 vCD3OD/CDCl3, mixing time 600 msec). Fig. S6. HSQC spectrum of 6-epi-alteramide A (1:1 CD3OD/CDCl3). Fig. S7. HSQC spectrum of 6-epi-alteramide B (1:1 CD3OD/CDCl3). Fig. S8. 1H NMR spectrum of 6-epi-alteramide B (CD3OD, 500 MHz). Fig. S9. Comparison 1H NMR spectra of 6-epi-alteramide A (blue) and 6-epi-alteramide B (red) (1:1 CD3OD/CDCl3, 500 MHz). Fig. S10. COSY spectrum of 6-epi-alteramide B (CD3OD). Fig. S11. TOCSY spectrum of 6-epi-alteramide B (CD3OD, mixing time 90 msec). Fig. S12. NOESY spectrum of 6-epi-alteramide B (CD3OD, mixing time 600 msec). Fig. S13. HSQC spectrum of 6-epi-alteramide B (CD3OD). Fig. S14. Expansion (olefinic region) of the HSQC spectrum of 6-epi-alteramide B (CD3OD). Assignments are indicated by numbers. Circled signals correspond to the cycloaddition product. Fig. S15. Expansion (aliphatic region) of the HSQC spectrum of 6-epi-alteramide B (CD3OD). Assignments are indicated by numbers. Table S1. Primer sets used in this work for the amplification of DNA regions used in gene inactivation and gene expression. Table S2. 6-epi-alteramide A 1H and 13C NMR data acquired in 1:1 CD3OD/CDCl3 (500 MHz, 24 °C). Table S3. 6-epi-alteramide A 1H and 13C NMR data acquired in CD3OD (500 MHz, 24 °C). Table S4. 6-epi-alteramide B 1H and 13C NMR data acquired in CD3OD (500 MHz, 24 °C). [file mbt20007-0242-sd1.doc]

**Activation and identification of five clusters for secondary metabolites in**

***Streptomyces albus* J1074**

Carlos Olano, Ignacio García, Aranzazu González, Miriam Rodriguez, Daniel Rozas, Julio Rubio, Marina Sánchez-Hidalgo, Alfredo F. Braña, Carmen Méndez and José A. Salas*

Running title: secondary metabolite clusters in *Streptomyces*

Departamento de Biología Funcional e Instituto Universitario de Oncología del Principado de Asturias (I.U.O.P.A), Universidad de Oviedo, 33006 Oviedo, Spain

* Corresponding author: Tel. +34-985 103652. E-mail: jasalas@uniovi.es

**Table S1.** Primer sets used in this work for the amplification of DNA regions used in gene inactivation and gene expression.

| **Primer** | **Sequence (5´-3´)** | **Description*** |
| --- | --- | --- |
| SA313A | AA**GGATCC**ATGCCGTGATGCCGTCTCGC | BamHI |
| SA313B | A**GAATTC**GAAGGGGTGGTGGTAGTAGAC | EcoRI |
| SA5712A | TT**CTGCAG**CATTTCCTGGGGGTGTTCTG | PstI |
| SA5712B | TT**TCTAGA**GATCCGGTTCGACACCATC | XbaI |
| SA5713A | AA**CTGCAG**CCACCTGAGGAGTGTGCGA | PstI |
| SA5713B | AA**TCTAGA**AGTGGACGACGGAGAGCC | XbaI |
| 11R1A1 | TT**GGATCC**ATGACGACCACGCCGCCC | BamHI |
| 11R1B1 | T**GAATTC**AGGCGAGGGCCGAGCG | EcoRI |
| 078FW | AC**GAATTC**CACCCTGTCACTGTGACC | EcoRI |
| 078RV | CG**AGATCT**CCGTAATGCGAAACGATG | BglII |
| SA5238FW | CATGAC**ACTAGT**CACGATCCGCACCTCGGAC | SpeI |
| SA5328RV | CTAGCA**GCATGC**CTCTACAGCGCTGGACGAG | SphI |
| SA5327FW | CATGAC**CATATG**CGCTGGTGCGGGACTCCGA | NdeI |
| SA5327RV | CTAGCA**GGATCC**GACGACGGCACGGTGGATC | BamHI |

*Restriction site in bold in primer sequence.

**Structural characterization of 6-*epi*-alteramides**

The HRMS data of the first compound confirmed a molecular formula of C_29_H_38_N_2_O_5_, accounting for alteramide B or an isomer of this molecule. Detailed analysis of 1D and 2D NMR spectra acquired in CD_3_OD and in a mixture of CDCl_3_/CD_3_OD (1:1) revealed significant differences in the chemical shifts of the signals of this compound and alteramide A, although the same connectivity between the different structural units was established by analysis of COSY and TOCSY experiments. The magnitude of the coupling constants measured for the olefinic protons confirmed an *E* configuration for the double bonds, leaving therefore a change in the absolute configuration of one or several chiral centers as the most plausible explanation for the structural change with respect to alteramide B. Analysis of the NOESY spectrum revealed key correlations between H13 and H5, H7b, H10, H11 and H15, between H6 and H4, H7a, H12, and H14, and between H8 and H7a, both H9 protons and H12. These correlations indicated an inversion of the configuration at C6 with respect to alteramide B, confirming therefore a structure of 6-*ep*i-alteramide B for this new compound. In a similar manner, a molecular formula of C_29_H_38_N_2_O_6_ determined by HRMS, and the analysis of NMR spectra, including key NOESY correlations measured in CDCl_3_/CD_3_OD (1:1) (Fig. S1), established a structure of 6-*ep*i-alteramide A for the second of the molecules isolated. The major differences in the spectra of this molecule and those of 6-epi-alteramide B were found in the replacement of the methylene at position C25 in 6-epi-alteramide B (*δ*_H_ 2.10, m and 1.87, m; *δ*_C_, 26.1) by an oxygenated methine in the structure of 6-*epi*-alteramide A ((*δ*_H_ 4.02,; *δ*_C_, 71.1), confirming thus the hydroxylation at this position. The absolute configuration of this new chiral center was assumed to be the same as in alteramide A.

**Fig. S1**. Key NOESY correlations observed in the structure of 6-*epi*-alteramide A.

**Fig. S2.** ^1^H NMR spectrum of 6-*epi*-alteramide A (CD_3_OD, 500 MHz).


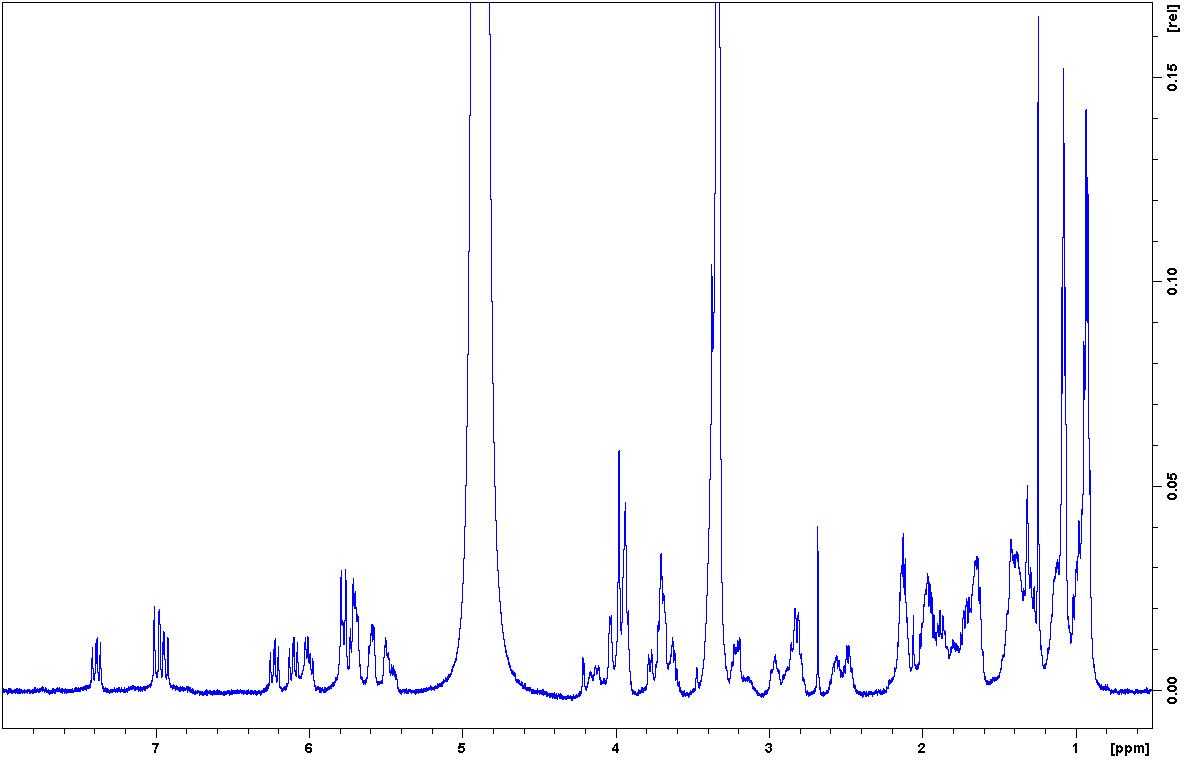


**Fig. S3.** ^1^H NMR spectrum of 6-*epi*-alteramide A (1:1 CD_3_OD/CDCl_3_, 500 MHz).


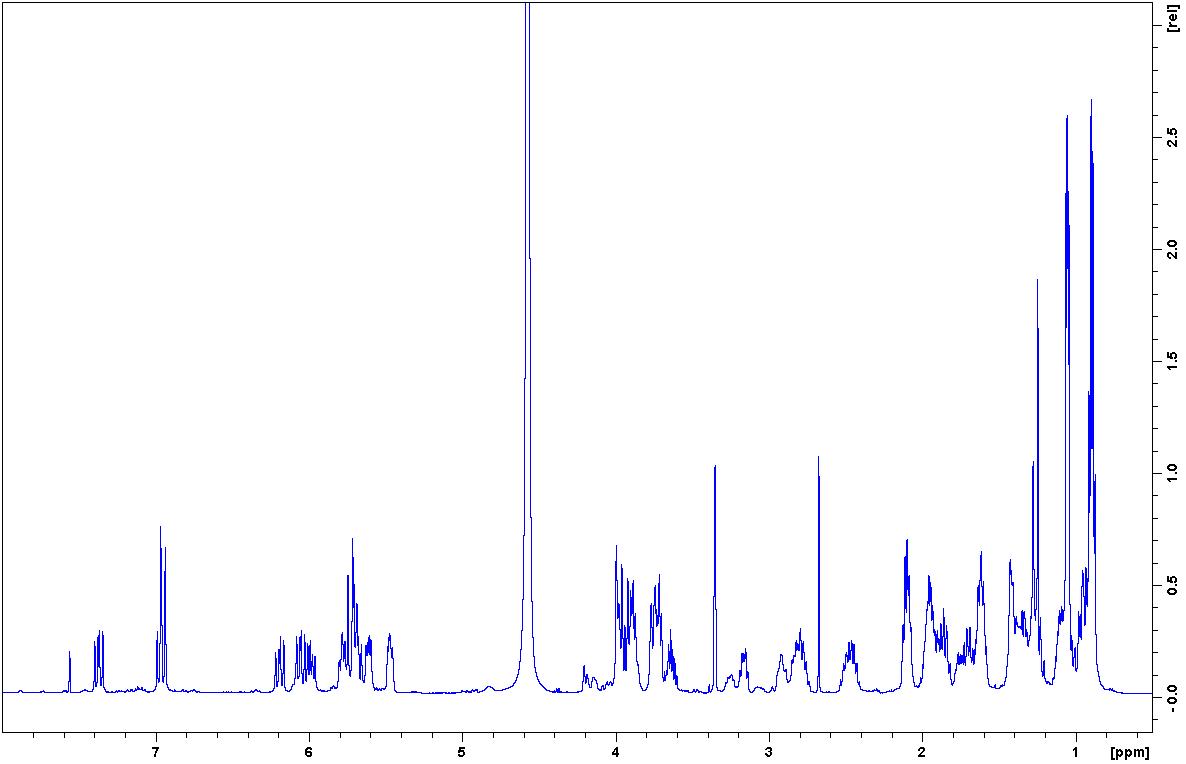


**Fig. S4**. COSY spectrum of 6-*epi*-alteramide A (1:1 CD_3_OD/CDCl_3_).


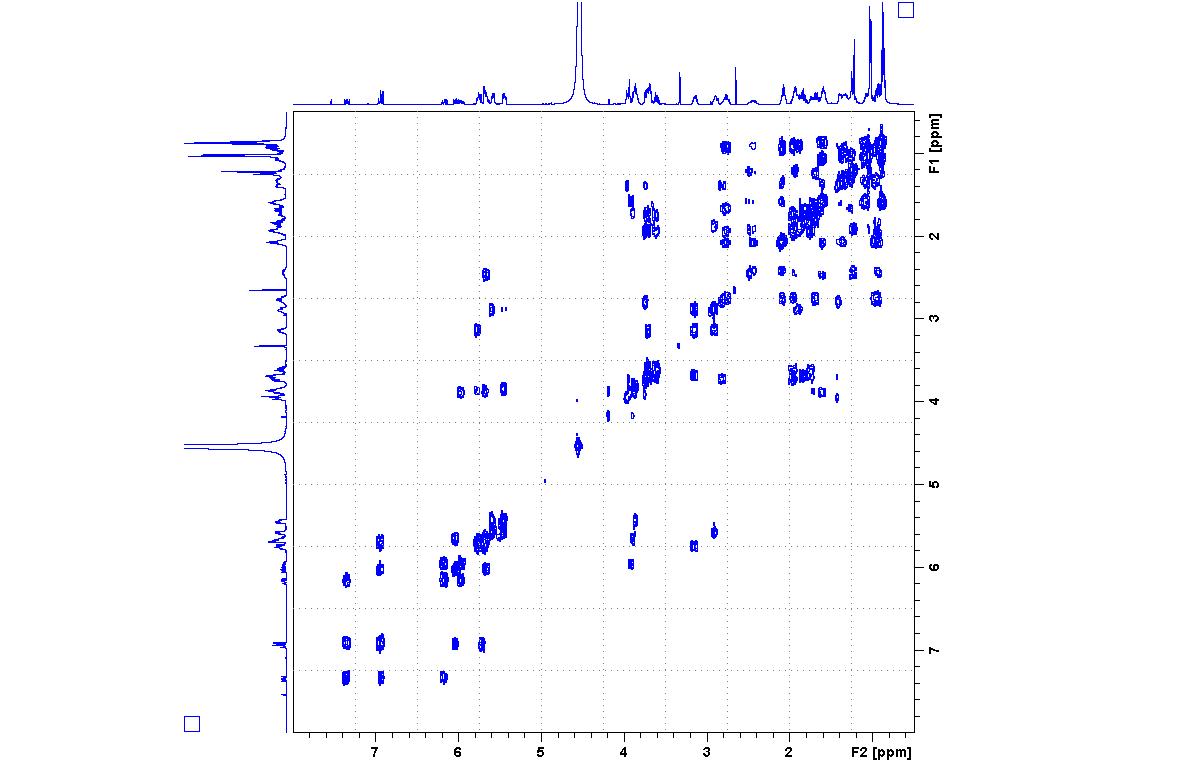


**Fig. S5.** NOESY spectrum of 6-*epi*-alteramide A (1:1 CD_3_OD/CDCl_3_, mixing time 600 msec).


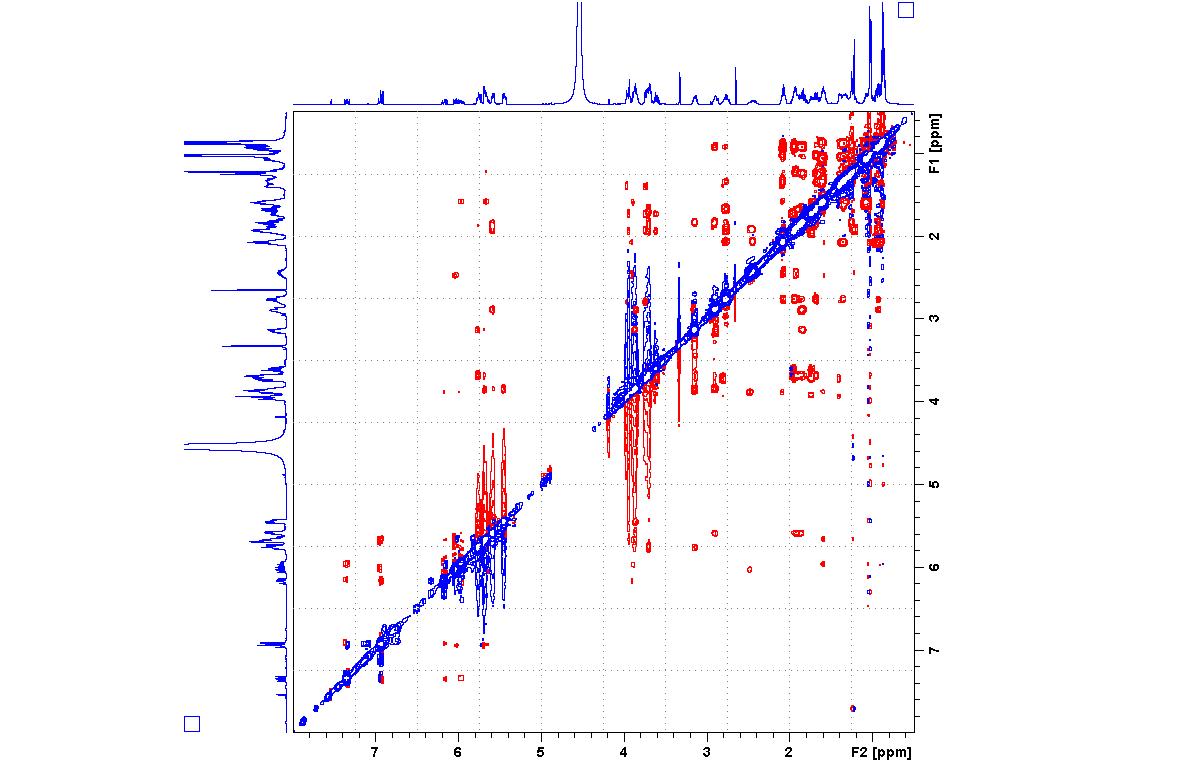


**Fig. S6.** HSQC spectrum of 6-*epi*-alteramide A (1:1 CD_3_OD/CDCl_3_).


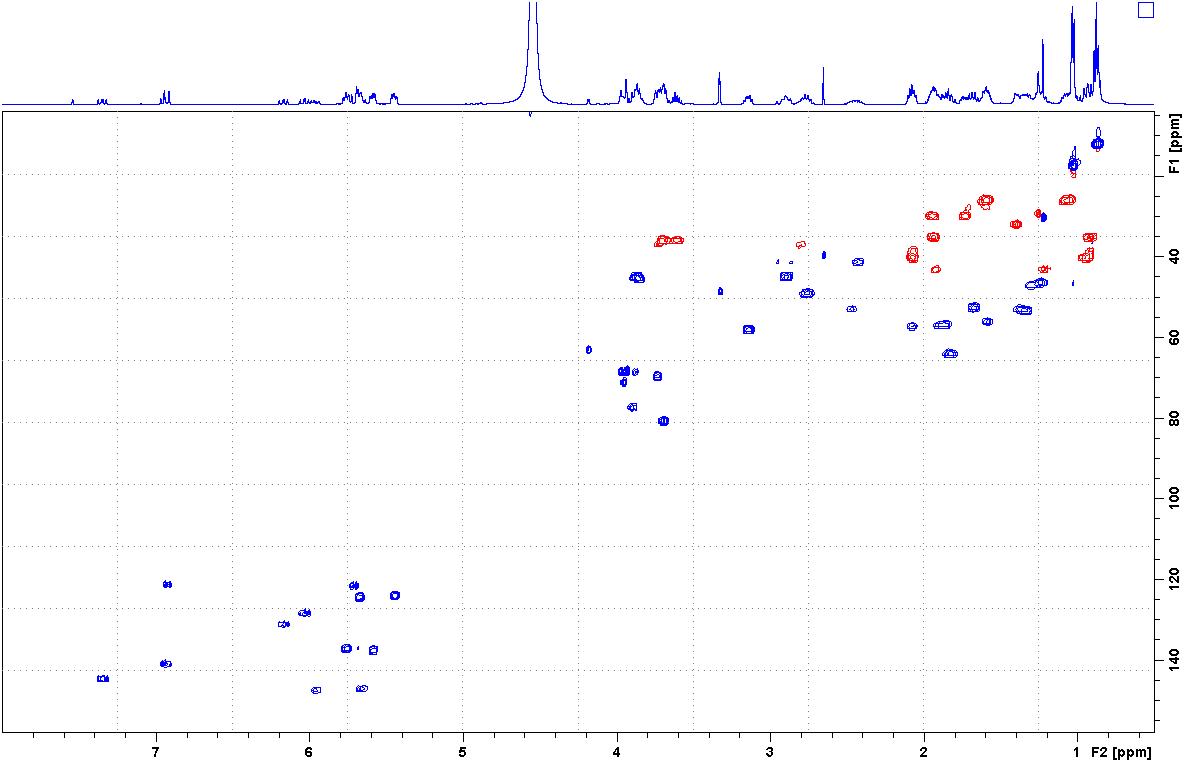


**Fig. S7.** HSQC spectrum of 6-*epi*-alteramide B (1:1 CD_3_OD/CDCl_3_).


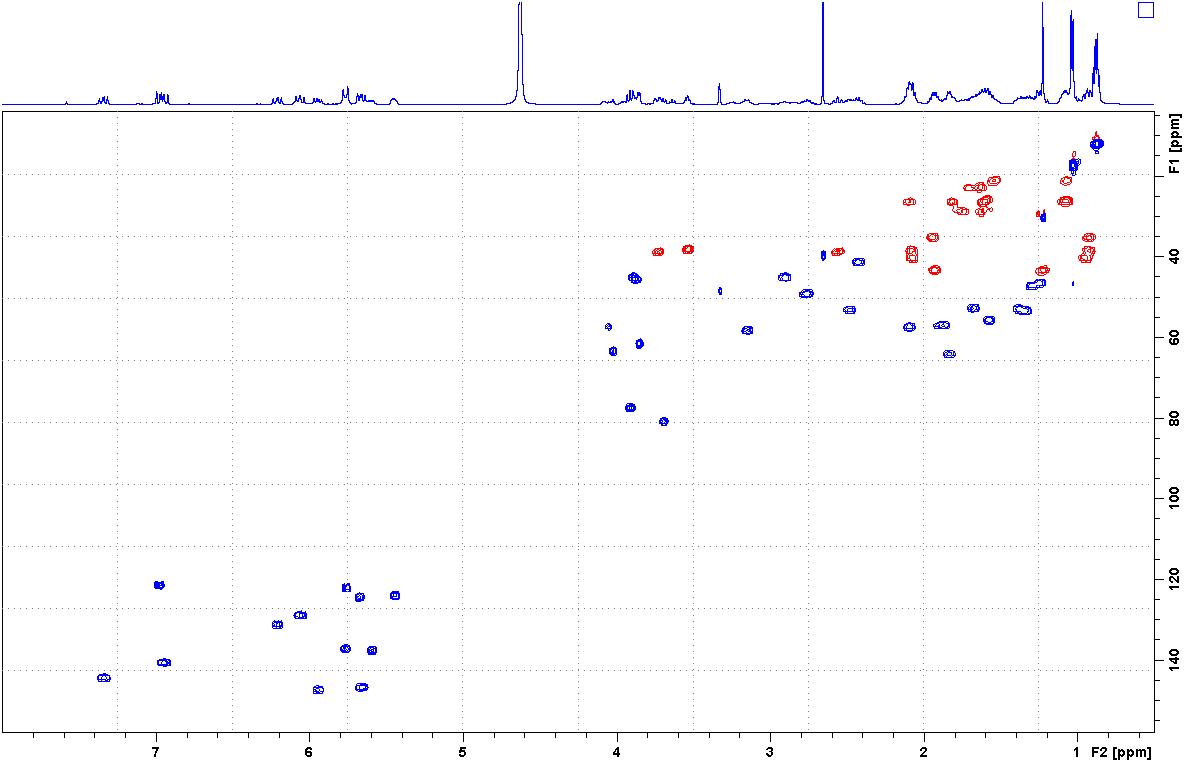


**Table S2.** 6-*epi*-alteramide A ^1^H and ^13^C NMR data acquired in 1:1 CD_3_OD/CDCl_3_ (500 MHz, 24 °C)

| **Position** | **^1^H** | ***J* (Hz)** | **^13^C** |
| --- | --- | --- | --- |
| 1 | - |  | n. d. |
| 2 | 5.71, d | 15.1 | 121.7 |
| 3 | 6.94, dd | 15.1, 11.1 | 141.0 |
| 4 | 6.03, dd | 14.9, 11.1 | 128.4 |
| 5 | 5.66, dd | 14.9, 9.5 | 147.2 |
| 6 | 2.47, m |  | 53.0 |
| 7 | (a) 1.92, m ; (b) 1.22, m |  | 43.1 |
| 8 | 2.43, m |  | 41.3 |
| 9 | (a) 2.08, m ; (b) 0.92, m |  | 38.5 |
| 10 | 1.38, m |  | 53.0 |
| 11 | 1.31, m |  | 47.0 |
| 12 | 2.08, m |  | 57.3 |
| 13 | 1.59, m |  | 56.0 |
| 14 | 3.90, m |  | 77.3 |
| 15 | 5.96, dd | 15.4, 8.2 | 147.6 |
| 16 | 6.17, dd | 15.4, 10.9 | 131.3 |
| 17 | 7.35, dd | 15.4, 10.9 | 144.6 |
| 18 | 6.93, d | 15.4 | 121.3 |
| 19 | - |  | n. d. |
| 20 | - |  | n. d. |
| 21 | - |  | n. d. |
| 22 | - |  | - |
| 23 | 3.97, br s |  | 68.5 |
| 24 | - |  | n. d. |
| 25 | 3.96, m |  | 71.1 |
| 26 | 1.40 m |  | 32.0 |
| 27 | (a) 3.72, m ; (b) 2.80, m |  | 37.0 |
| 28 | - |  | - |
| 29 | (a) 1.59, m ; (b) 1.07, m |  | 26.0 |
| 30 | 0.88, t | 7.0 | 12.1 |
| 31 | 1.02, d | 6.5 | 17.3 |

(n. d. = not determined; ^13^C NMR frequencies were determined from the HSQC spectrum).

**Table S3.** 6-*epi*-alteramide A ^1^H and ^13^C NMR data acquired in CD_3_OD (500 MHz, 24 °C)

| **Position** | **^1^H** | ***J* (Hz)** | **^13^C** |
| --- | --- | --- | --- |
| 1 | - |  | n. d. |
| 2 | 5.81, d | 15.1 | 121.6 |
| 3 | 6.94, dd | 15.1, 11.1 | 140.6 |
| 4 | 6.12, dd | 14.9, 11.1 | 128.8 |
| 5 | 5.69, dd | 14.9, 9.5 | 147.0 |
| 6 | 2.56, m |  | 53.0 |
| 7 | (a) 1.94, m ; (b) 1.26, m |  | 43.0 |
| 8 | 2.47, m |  | 41.3 |
| 9 | (a) 2.12, m ; (b) 0.96, m |  | 38.5 |
| 10 | 1.40, m |  | 53.1 |
| 11 | 1.35, m |  | 47.4 |
| 12 | 2.13, m |  | 57.4 |
| 13 | 1.61, m |  | 55.9 |
| 14 | 3.93, m |  | 77.3 |
| 15 | 5.99, dd | 15.4, 8.2 | 147.2 |
| 16 | 6.22, dd | 15.4, 10.9 | 131.4 |
| 17 | 7.38, dd | 15.4, 10.9 | 143.9 |
| 18 | 6.99, d | 15.4 | 121.5 |
| 19 | - |  | n. d. |
| 20 | - |  | n. d. |
| 21 | - |  | n. d. |
| 22 | - |  | - |
| 23 | 3.98, br s |  | 68.4 |
| 24 | - |  | n. d. |
| 25 | 4.02, m |  | 71.1 |
| 26 | 1.42 m |  | 31.5 |
| 27 | (a) 3.68, m ; (b) 2.82, m |  | 37.2 |
| 28 | - |  | - |
| 29 | (a) 1.65, m ; (b) 1.12, m |  | 26.0 |
| 30 | 0.92, t | 7.0 | 11.6 |
| 31 | 1.07, d | 6.5 | 16.9 |

(n. d. = not determined; ^13^C NMR frequencies were determined from the HSQC spectrum).

**Fig. S8.** ^1^H NMR spectrum of 6-*epi*-alteramide B (CD_3_OD, 500 MHz).


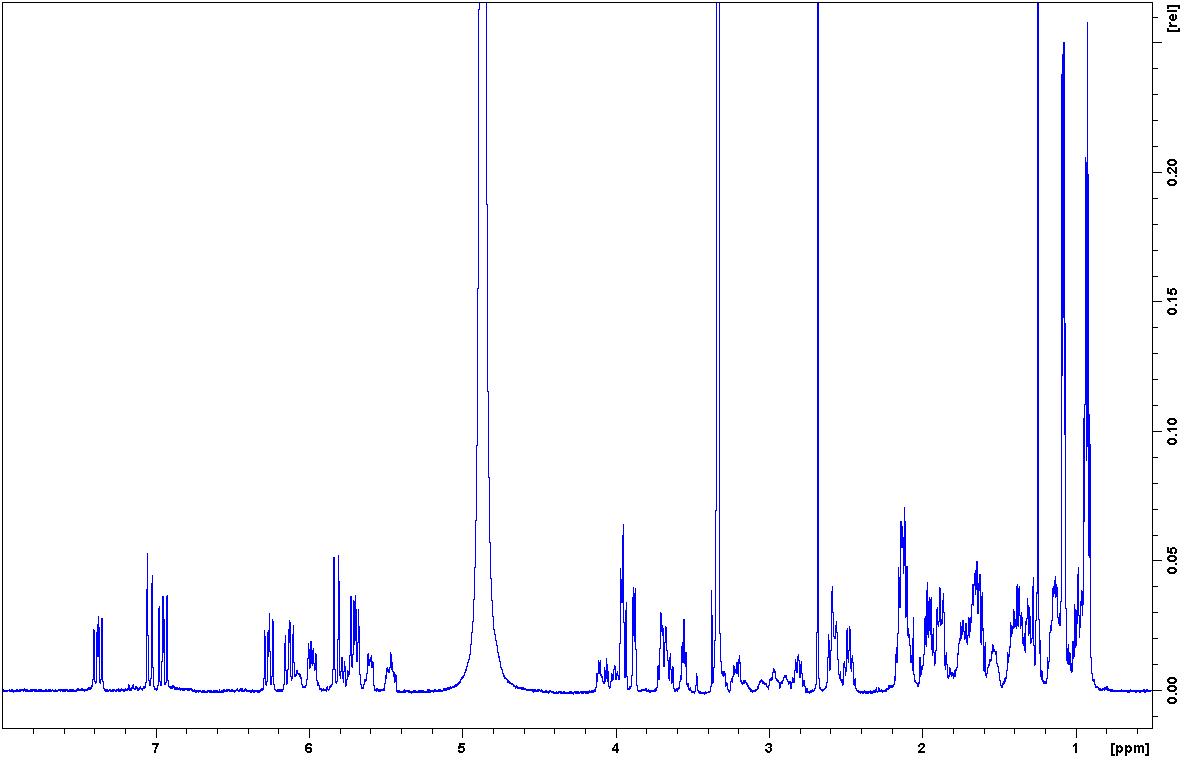


**Fig. S9.** Comparison ^1^H NMR spectra of 6-*epi*-alteramide A (blue) and 6-*epi*-alteramide B (red) (1:1 CD_3_OD/CDCl_3_, 500 MHz).


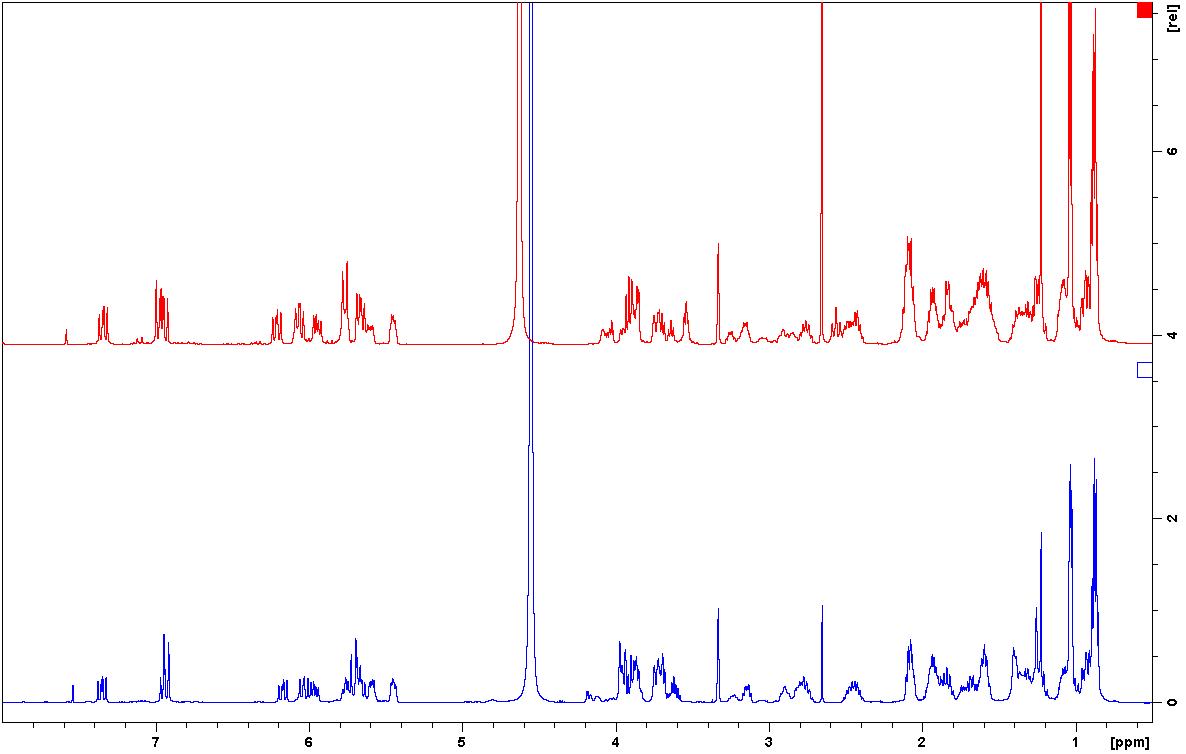


**Fig. S10.** COSY spectrum of 6-*epi*-alteramide B (CD_3_OD).


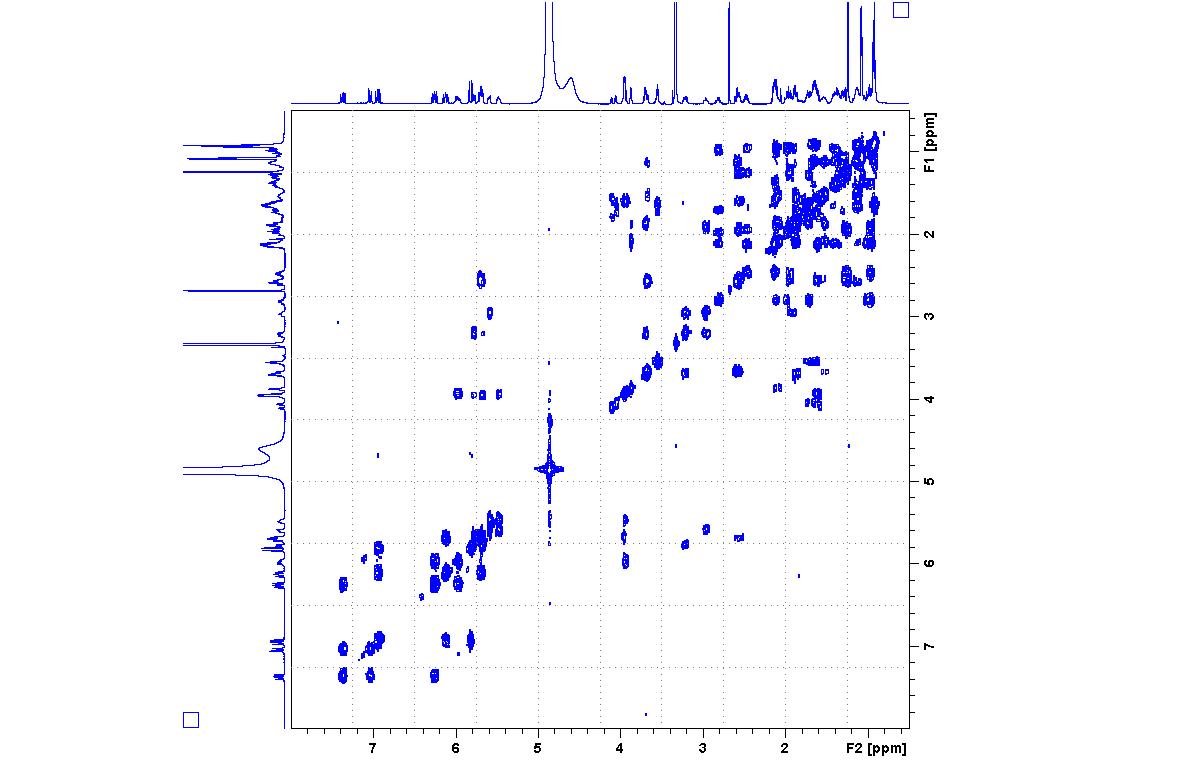


**Fig. S11.** TOCSY spectrum of 6-*epi*-alteramide B (CD_3_OD, mixing time 90 msec).


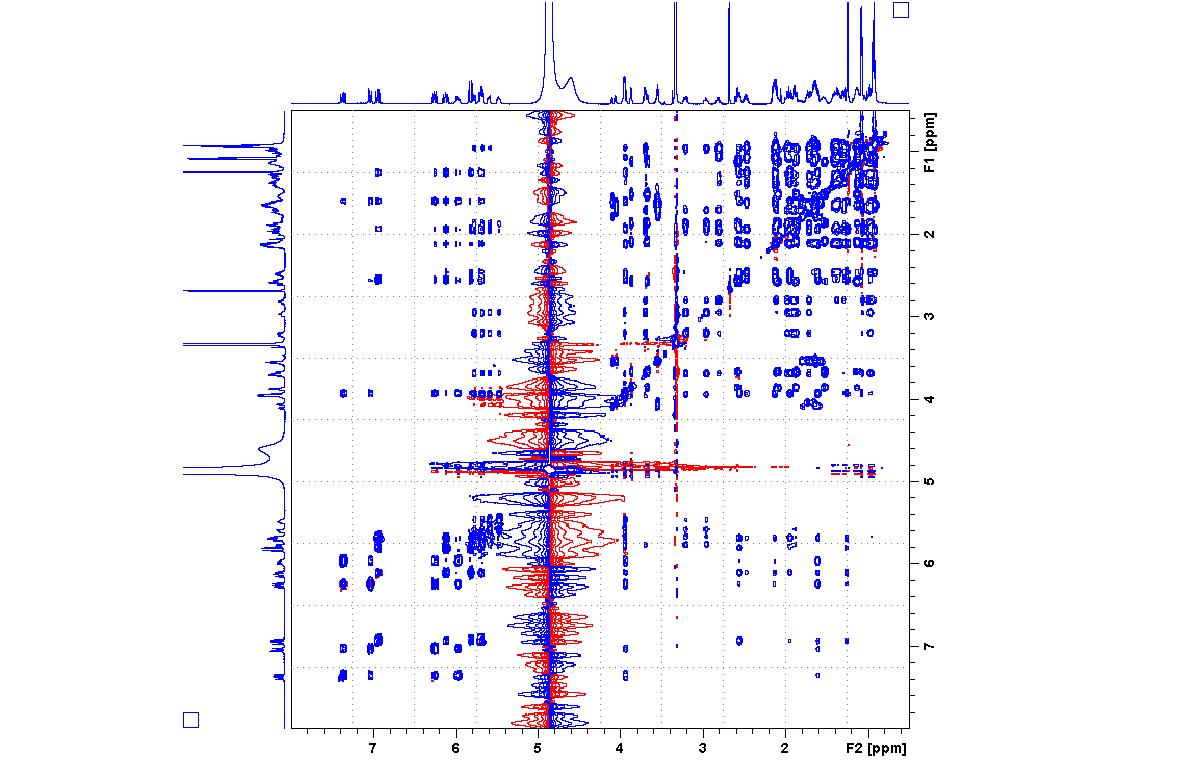


.

**Fig. S12.** NOESY spectrum of 6-*epi*-alteramide B (CD_3_OD, mixing time 600 msec).


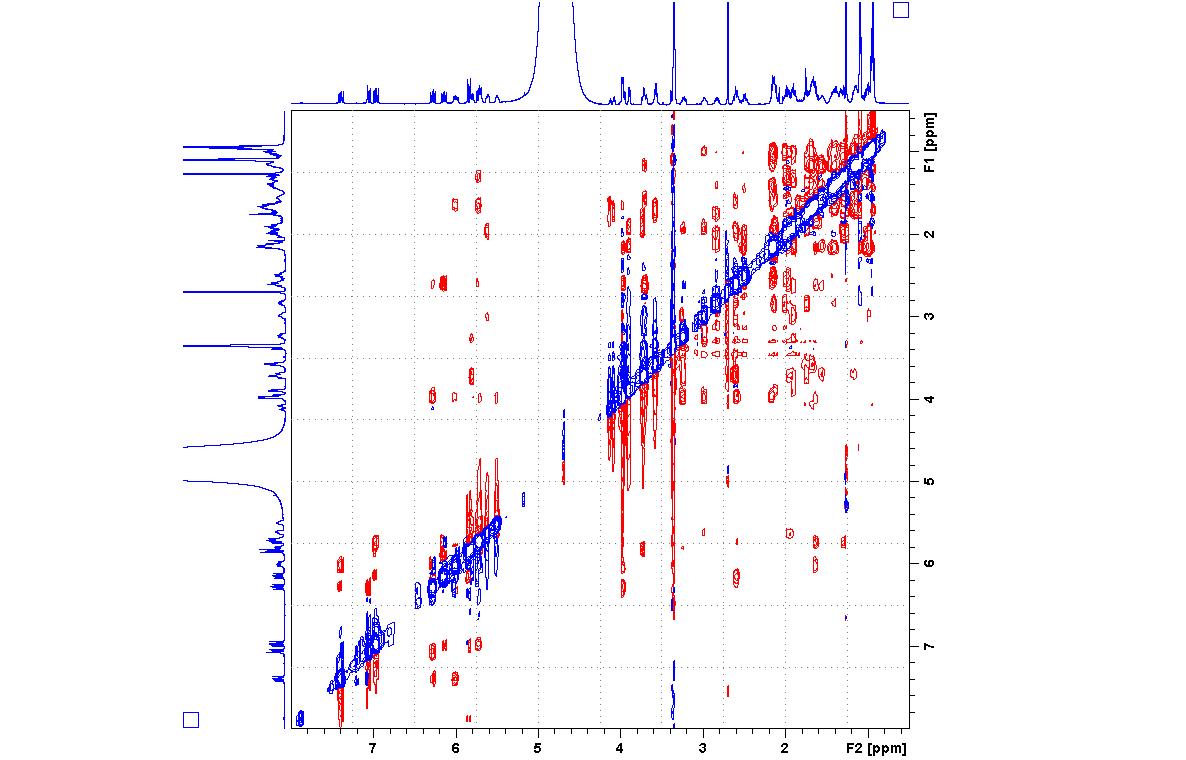


**Fig. S13.** HSQC spectrum of 6-*epi*-alteramide B (CD_3_OD).


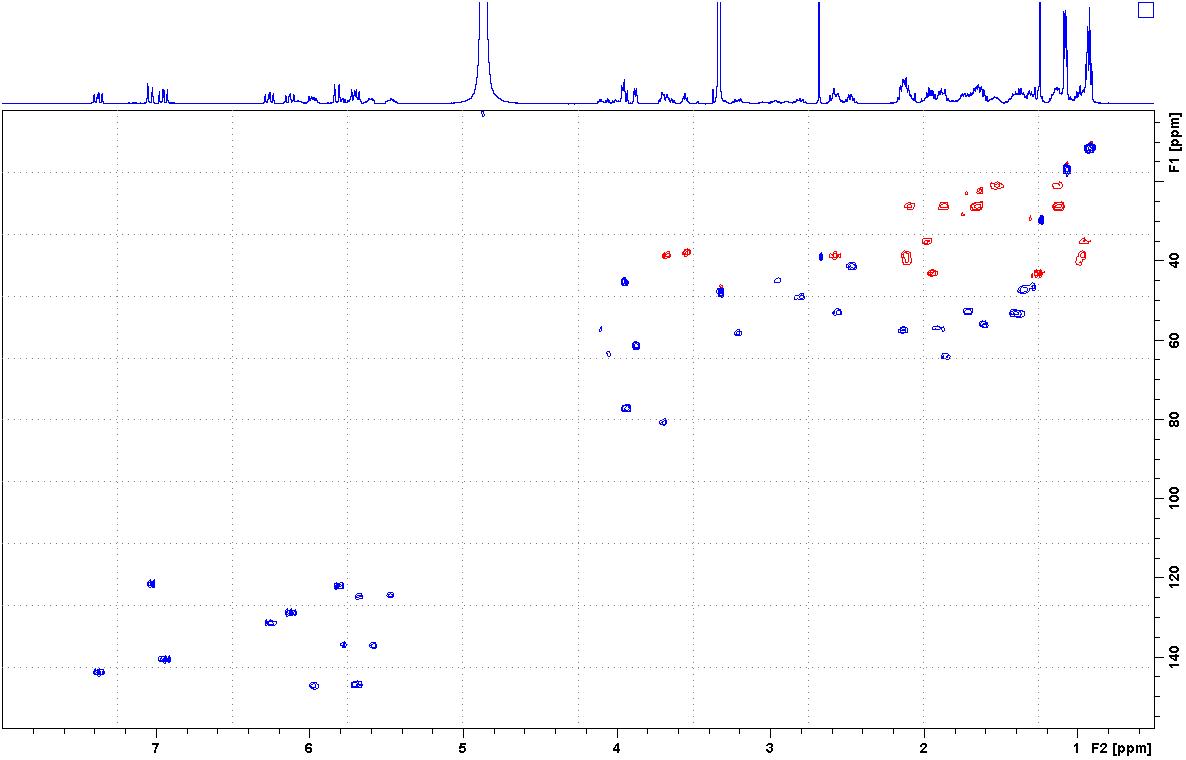


**Fig. S14.** Expansion (olefinic region) of the HSQC spectrum of 6-*epi*-alteramide B (CD_3_OD). Assignments are indicated by numbers. Circled signals correspond to the cycloaddition product.


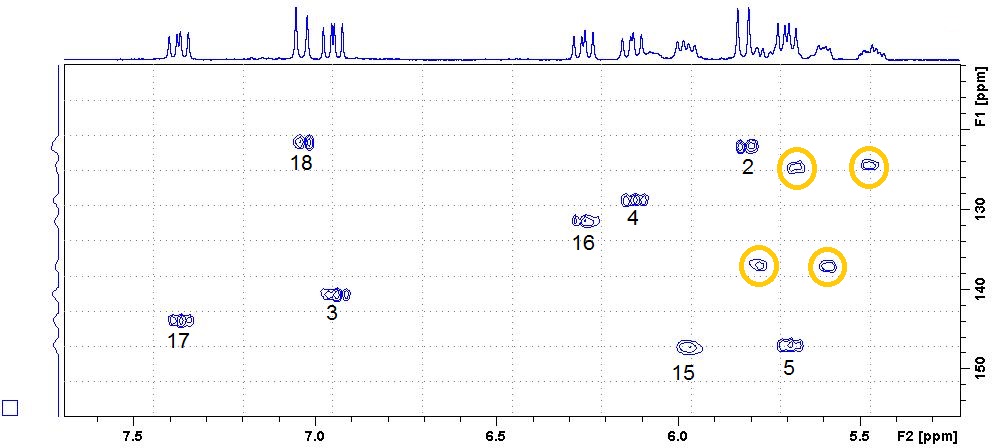


**Fig. S15.** Expansion (aliphatic region) of the HSQC spectrum of 6-*epi*-alteramide B (CD_3_OD). Assignments are indicated by numbers.


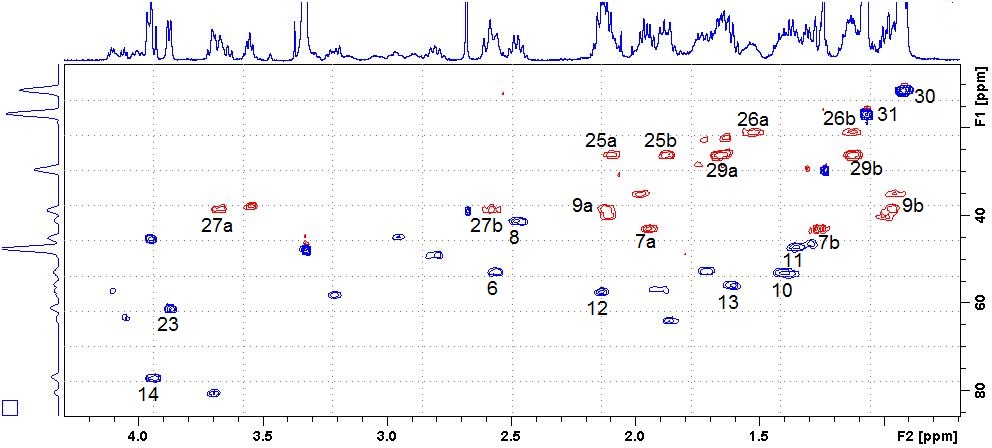


**Table S4.** 6-*epi*-alteramide B ^1^H and ^13^C NMR data acquired in CD_3_OD (500 MHz, 24 °C)

| **Position** | **^1^H** | ***J* (Hz)** | **^13^C** |
| --- | --- | --- | --- |
| 1 | - |  | n. d. |
| 2 | 5.77, d | 15.1 | 122.0 |
| 3 | 6.95, dd | 15.1, 11.1 | 141.1 |
| 4 | 6.10, dd | 14.9, 11.1 | 128.8 |
| 5 | 5.69, dd | 14.9, 9.5 | 147.6 |
| 6 | 2.55, m |  | 53.2 |
| 7 | (a) 1.95, m ; (b) 1.24, m |  | 43.2 |
| 8 | 2.48, m |  | 41.4 |
| 9 | (a) 2.11, m ; (b) 0.97, m |  | 39.6 |
| 10 | 1.39, m |  | 53.4 |
| 11 | 1.35, m |  | 47.2 |
| 12 | 2.14, m |  | 57.4 |
| 13 | 1.61, m |  | 55.9 |
| 14 | 3.94, m |  | 77.3 |
| 15 | 5.97, dd | 15.4, 8.2 | 147.2 |
| 16 | 6.25, dd | 15.4, 10.9 | 131.3 |
| 17 | 7.37, dd | 15.4, 10.9 | 143.8 |
| 18 | 7.03, d | 15.4 | 121.5 |
| 19 | - |  | n. d. |
| 20 | - |  | n. d. |
| 21 | - |  | n. d. |
| 22 | - |  | - |
| 23 | 3.87, d | 6.0 | 61.4 |
| 24 | - |  | n. d. |
| 25 | (a) 2.10, m ; (b) 1.87, m |  | 26.1 |
| 26 | (a) 1.52, m ; (b) 1.13, m |  | 21.0 |
| 27 | (a) 3.68, m ; (b) 2.58, m |  | 38.6 |
| 28 | - |  | - |
| 29 | (a) 1.66, m ; (b) 1.12, m |  | 26.2 |
| 30 | 0.92, t | 7.0 | 11.5 |
| 31 | 1.07, d | 6.5 | 16.8 |

(n. d. = not determined; ^13^C NMR frequencies were determined from the HSQC spectrum).
